# ^99^TcO_4_^−^ removal from legacy defense nuclear waste by an alkaline-stable 2D cationic metal organic framework

**DOI:** 10.1038/s41467-020-19374-9

**Published:** 2020-11-04

**Authors:** Nannan Shen, Zaixing Yang, Shengtang Liu, Xing Dai, Chengliang Xiao, Kathryn Taylor-Pashow, Dien Li, Chuang Yang, Jie Li, Yugang Zhang, Mingxing Zhang, Ruhong Zhou, Zhifang Chai, Shuao Wang

**Affiliations:** 1 State Key Laboratory of Radiation Medicine and Protection, School for Radiological and Interdisciplinary Sciences (RAD-X) and Collaborative Innovation Center of Radiation Medicine of Jiangsu Higher Education Institutions, Soochow University, Suzhou, 215123 China; 2 College of Chemical and Biological Engineering, Zhejiang University, Hangzhou, 310027 China; 3 Savannah River National Laboratory, Aiken, SC 29808 USA

**Keywords:** Pollution remediation, Solid-state chemistry, Nuclear chemistry

## Abstract

Removal of ^99^TcO_4_^−^ from legacy defense nuclear tank waste at Savannah River Site is highly desirable for the purpose of nuclear safety and environmental protection, but currently not achievable given the extreme conditions including high alkalinity, high ionic strength, and strong radiation field. Herein, we present a potential solution to this long-term issue by developing a two-dimensional cationic metal organic framework SCU-103, showing ultrahigh stability in alkaline aqueous media and great resistance to both β and γ radiation. More importantly, it is very effective for ^99^TcO_4_^−^ separation from aqueous media as demonstrated by fast exchange kinetics, high sorption capacity, and superior selectivity, leading to the successful removal of ^99^TcO_4_^−^ from actual Savannah River Site high level tank waste for the first time, to the best of our knowledge. In addition, the uptake mechanism is comprehensively elucidated by molecular dynamics simulation and density functional theory calculation, showing a unique chemical recognition of anions with low charge density.

## Abstract (web summary)

Separation of ^99^TcO_4_^−^ from nuclear waste at the Savannah River Site is hampered by the extreme conditions. Here, the authors propose a solution by developing an alkaline-resistant metal organic framework material featuring unique recognition sites for selective incorporation of ^99^TcO_4_^−^ anions.

## Introduction

With the development of nuclear power technology, radioactive waste treatment and contamination remediation are great challenges. The Savannah River Site (SRS), located along the Savannah River in western South Carolina (US), was built in the 1950s with the primary mission to produce tritium and plutonium for national defense programs^1^. The main responsibilities of SRS at present are environmental restoration and remediation. Up to date, the vast bulk of legacy nuclear wastes composed of sludge and supernatant liquid that have been generated are stored in underground tanks at SRS awaiting pretreatment and safe disposal^2,3^. Among these, ^99^Tc, a long-lived β-emitting radionuclide, is considered to be one of the most problematic radionuclides. With a high fission yield of 6%, ~400 metric tonnes of ^99^Tc have been produced since the utilization of the first nuclear reactor^4,5^. At present, a large inventory of ^99^Tc, which exists primarily in its most stable oxidation state as the pertechnetate anion (TcO_4_^−^) under aerobic environments, is present in the high-level waste (HLW) at SRS^6^. Generally, much of the ^99^Tc at SRS is cast into a cementitious low-level waste form, referred to as “saltstone”, whereas at the Hanford site, both the high and low-level fractions will be immobilized into a borosilicate glass waste form (a process known as vitrification)^7^. Considering the volatile nature of Tc(VII) complexes during the high temperature vitrification processes, there is a crucial need for an effective strategy for ^99^TcO_4_^−^ removal from nuclear waste prior to vitrification. In addition, the non-complexing nature and low charge density of ^99^TcO_4_^−^ give rise to its high solubility (11.3 mol/L at 20 °C) in water and subsequently high environmental mobility with a migration velocity similar to groundwater flow on the subsurface^4^. At both the Hanford and SRS, ^99^Tc has been released into the subsurface environment, resulting in concentrations in the groundwater at least one order of magnitude higher than the maximum contaminant level established by the US EPA (900 pCi/L)^8^. Therefore, it is highly desirable and urgent to remove ^99^TcO_4_^−^ from HLW at SRS for both nuclear waste management and environmental remediation. However, this is extremely challenging given the extreme conditions of super alkalinity, high salinity, and strong ionizing radioactivity in HLW streams^3,7^.

Considering the anionic nature of ^99^TcO_4_^−^, tremendous efforts have been devoted to developing cationic framework materials as ^99^TcO_4_^−^ scavengers owing to strong targeted electrostatic attraction or host–guest interaction. To date, several types of cationic materials including purely inorganic cationic frameworks, anion-exchange resins, cationic polymeric networks (CPNs), cationic covalent organic frameworks (COFs), and cationic metal organic frameworks (MOFs) have been tested for ^99^TcO_4_^−^ separation capability from nuclear waste solutions. For inorganic cationic materials such as layered double hydroxides (LDHs)^9,10^, Y_2_(OH)_5_Cl^11^, Yb_3_O(OH)_6_Cl^12^, NDTB-1^13,14^, and metal sulfides^15^, poor selectivity severely limits their practical applications in actual nuclear waste streams at SRS since a large number of competitive anions including NO_3_^−^, SO_4_^2−^, CO_3_^2−^, PO_4_^3−^, and OH^−^ coexist with ^99^TcO_4_^−^ in excess of 100 to 6000 fold. Purely inorganic anion-exchange materials also suffer from low capacity and a narrow applicable pH range^16^. Commercially available anion-exchange resins deliver high uptake selectivity towards ^99^TcO_4_^−^^17,18^, however, poor radiation-resistance results in gradual decrease in uptake capacity with the increase of exposed radiation dosage during the anion-exchange process^19^. In addition, the sorption kinetics are rather slow and the elution is difficult owing to the strong affinity between resins and ^99^TcO_4_^−^ anion. The recently developed cationic COF^20^ and CPN^21,22^ overcome the shortcomings of the aforementioned materials, and possess remarkable advantages in ^99^TcO_4_^−^ separation under highly acidic conditions (e.g., 3 M nitric acid) that are found in used fuel reprocessing. However, these cationic materials exhibit notably poor alkaline stability because the cationic pyridine or imidazolium ring undergoes ring-opening reactions induced by nucleophilic attack from OH^−^ in alkaline solutions. Therefore, a qualified ^99^TcO_4_^−^ capture material with combined characteristics of excellent alkaline stability, radiation resistance, and excellent selectivity aiming for legacy defense waste partitioning at SRS currently remains elusive.

Metal organic frameworks (MOFs), assembled by organic linkers and inorganic metal ions or clusters, have evolved as an important branch of porous functional materials^23,24^. Considering the diversity of inorganic and organic components, MOFs exhibit versatile structures and properties, giving them great potential in multiple applications, including gas storage^25,26^, adsorption/separation^27^, luminescence^28^, and catalysis^29,30^. More significantly, MOFs have been proven to be powerful in the field of environmental remediation, especially in the sequestration of toxic pollutants from aqueous solutions via adsorption or ion-exchange processes^31^. As a subclass, cationic MOFs constructed by neutral nitrogen-containing ligands and metal ions have attracted significant research attention as anion-exchange hosts owing to the presence of substitutable uncoordinated anions residing in the void spaces^32–36^. Compared with traditional cationic porous materials, MOFs afford some clear advantages. In particular, the highly tunable structures, achievable by rational selections of inorganic and organic components, or via post-synthetic modification of the surface, contributes greatly to the enhancement of separation selectivity. Furthermore, the crystalline nature allows for precise structure–property correlation, which is greatly beneficial for identifying the separation/exchange mechanism. To date, several types of oxo-anions including CrO_4_^2−^, Cr_2_O_7_^2−^, MnO_4_^−^ were reported to be captured by cationic MOFs^37–41^. A series of cationic MOFs as ReO_4_^−^/^99^TcO_4_^−^ scavengers with high uptake capacity, fast kinetics, excellent sorption selectivity, and good radiation resistance have also been developed recently^16,42–49^. Despite these advantages, the relatively low hydrolytic and chemical stability has stalled the progress of these materials for real-time TcO_4_^−^ separation applications, especially under extremely alkaline conditions.

Therefore, we sought to develop alkaline-resistant cationic MOFs for ^99^TcO_4_^−^ segregation in SRS HLW streams. Based upon the hard-soft-acid-base theory^50^, the combination of carboxylate groups and high-valent metal ions such as Zr^4+^, Hf^4+^, Fe^3+^, Al^3+^, Cr^3+^, which are characterized as hard Lewis bases and acids, respectively, can facilitate the formation of strong coordination bonds with each other, furnishing frameworks with high stability in neutral aqueous solutions or even in concentrated acids^51–54^, whereas these materials usually undergo decomposition in alkaline conditions. The stability of MOFs is closely related to the robustness of the coordination bonds between metal ions and organic ligands and the disintegration of structure was attributed to the competitive coordination to metal ions between organic ligands and other molecular species or anions^55,56^. For the MOFs mentioned above, carboxylate groups coordinated to high-valent metal ions could be easily replaced by OH^−^ anions owing to their strong affinity toward high-valent metal ions, thus leading to decomposition in alkaline solutions. Therefore, aiming for decent alkaline stability, metal ions and organic ligands with enhanced softness are highly desirable. With this strategy, the binding interaction between OH^−^ anions and metal ions is dramatically weakened and the coordination bonds between metal ions and linkers are robust enough to resist the competition from OH^−^ anions^55^. This is particularly true for MOFs built from transition metal ions and nitrogen heterocyclic ligand with high *pK*a values^57^. However, the systematic design and development of cationic MOFs with good alkaline resistance is still in its infancy^35^.

Herein, we developed a two-dimensional cationic MOF by employing a neutral tridentate nitrogen ligand tris[4-(1H-imidazol-1-yl)-phenyl]amine (tipa) and Ni^2+^ ions, namely [Ni(tipa)_2_](NO_3_)_2_ (SCU-103). It overcomes the disadvantage of poor alkaline stability of reported cationic MOF materials and exhibits exceptional ^99^TcO_4_^−^ capture selectivity, leading to the successful separation of ^99^TcO_4_^−^ from actual legacy nuclear waste at SRS.

## Results

### Synthesis and structure description

**Fig. 1:**
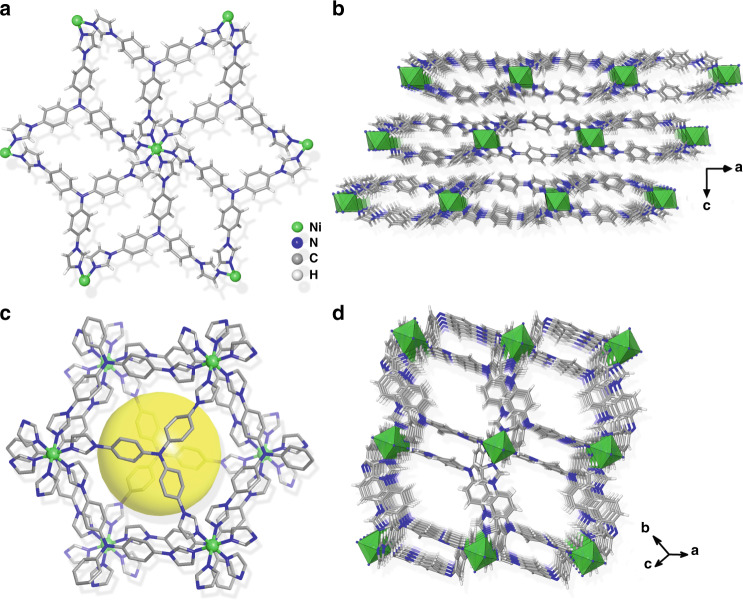
Crystal structure of SCU-103. **a** Coordination environment of Ni^2+^ ion with six ligands. **b** The packing diagram of 2D layers in SCU-103. **c** Zoomed view of the cavity formed by the face to face coupling of concave–convex 2D layers. **d** Perspective view of packing in SCU-103 showing porous channels.

The facile synthesis of SCU-103 could be realized through a one-pot solvothermal route by reacting tipa ligand and Ni(NO_3_)_2_·6H_2_O in a mixture of N,N-dimethylformamide (DMF) and deionized water at 140 °C for 3 days. Single-crystal X-ray diffraction analyses reveal that SCU-103 crystallizes in a trigonal crystal system with the *R-*3 space group. The crystallographic asymmetric unit consists of one-sixth of a Ni^2+^ ion, one-third of the tipa ligand, as well as disordered nitrate anions and guest solvents. Each Ni^2+^ is in a six-coordinated octahedral geometry, defined by N atoms from six independent trigonal tipa ligands (Fig. 1a), extending the structure into 2D cationic layers along the *ab* plane. Topologically, by simplifying the ligands and Ni^2+^ cations as three and six connecting nodes, respectively, the cationic layer features a binodal (3,6)-connected kagomé dual (*kgd*) net topology with the Schläfli symbol of (4^3^)_2_(4^6^·6^6^·8^3^) (Supplementary Figure 1). An important characteristic of this topology is its reluctance to interpenetration, leading to potential porosity. In the present case, three coordinating arms in the trigonal ligand are featured with a significant “kink” in order to match the octahedral geometry of the metal center. If a plane across all Ni^2+^ atoms in the same cationic layer is defined, the tipa ligands are coordinated to the metal center in two orientations: half of them are located above the plane, whereas the others are below it. Such up–down alternate arrangement results in the formation of bowl-shaped voids (Supplementary Figure 2a) above each tipa ligand. The concave–convex 2D layers are packed in parallel in the *ABC* fashion along the *c* direction, as shown in Fig. 1b. The face to face adjacent bowls are coupled into a capsule (Fig. 1c) with the size estimated by Ni···Ni separation (~16.1 × 16.1 × 16.1 Å) and the distance between two adjacent N3 atoms (~12.9 Å) (Supplementary Figure 2b). The charge balancing nitrate anions and solvent molecules are located in the capsules. However, these anions and solvent molecules are completely disordered and cannot be recognized by diffraction data. In addition, such capsules are interconnected through spacious windows (~10.4 × 16.1 Å) (Fig. 1d), thus allowing guest molecules to transport efficiently.

### Hydrolytic and radiolytic stability

**Fig. 2:**
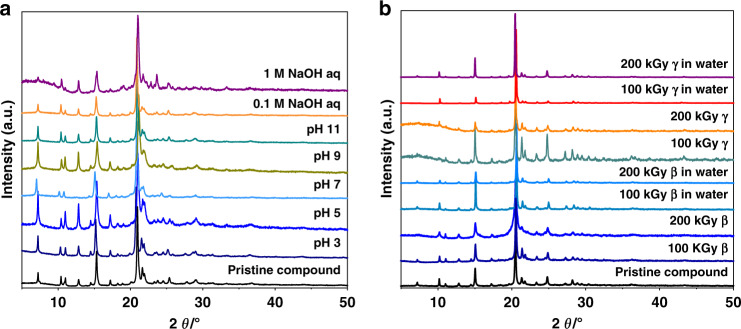
Powder X-ray diffraction of SCU-103. **a** PXRD patterns of SCU-103 after immersion in aqueous solutions with different pH values ranging from 3 to 14. **b** PXRD patterns of SCU-103 after β and γ radiation.

Except for some preferred orientation, the experimental powder X-ray diffraction (PXRD) pattern is basically consistent with the one simulated from single-crystal X-ray structure analyses, indicating the phase purity of SCU-103 (Supplementary Figure 3). Significantly, SCU-103 possesses great hydrolytic stability as the PXRD patterns after immersion into aqueous solutions with pH values ranging from 3 to 14 match well with those of the pristine compounds (Fig. 2a). Such a unique property is closely related to its structural characterizations. As depicted by the space-filling mode (Supplementary Figure 4), the metal centers are sterically crowded and enclosed within the concave–convex layers by non-planar tipa ligands, thus endowing SCU-103 with strong resistance to H_2_O and OH^−^ attack even under basic conditions. PXRD patterns of dried crystals and crystals immersed in water after 100 and 200 kGy of β and γ radiation remain almost identical to those of the original products (Fig. 2b). In addition, anion-exchange experiments performed after β and γ irradiation suggest no decrease in adsorption capacity of ReO_4_^−^ as compared with the original samples, further confirming excellent radiation resistance of SCU-103 (Fig. 4d, Supplementary Table 16). The excellent radiation resistance originates from the relatively large conjugated structure of the tipa ligand that can stabilize the radiation induced radicals, similar to several radiation resistant MOFs recently developed^58^. These results demonstrate that SCU-103 possesses sufficient robustness required for practical applications in high-level nuclear waste streams.

### Sorption kinetics analysis

**Fig. 3:**
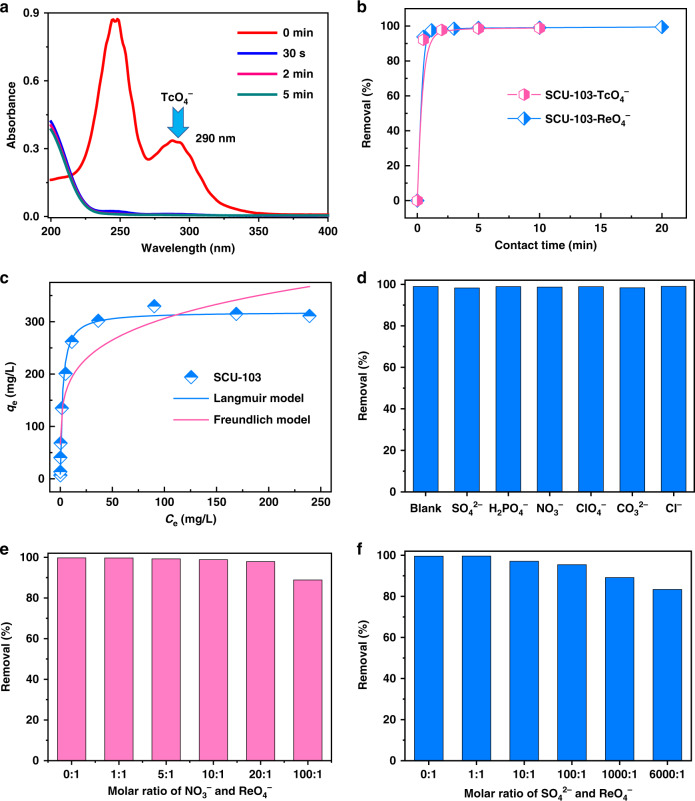
^99^TcO_4_^−^/ReO_4_^−^ sorption experiment results using SCU-103. **a** UV−vis spectra of ^99^TcO_4_^−^ solution during the anion-exchange with SCU-103. **b** Sorption kinetics of ^99^TcO_4_^−^ and ReO_4_^−^ by SCU-103. **c** Sorption isotherm of ReO_4_^−^ by SCU-103 (blue line: Langmuir model, pink line: Freundlich model). **d** Effects of different competing anions on the removal of ReO_4_^−^ by SCU-103. **e** Effect of excess NO_3_^−^ on the ReO_4_^−^ exchange. **f** Effect of excess SO_4_^2−^ on the ReO_4_^−^ exchange.

The anion-exchange experiment was initially performed by soaking 20 mg of SCU-103 samples in 20 mL of aqueous solution containing 14 ppm of ^99^TcO_4_^−^, in which the molar ratio of SCU-103 to ^99^TcO_4_^−^ was ~6:1. The effect of contact time of ^99^TcO_4_^−^ with SCU-103 was investigated to evaluate the anion-exchange rate and equilibrium time. As shown in Fig. 3a, the concentration of ^99^TcO_4_^−^ as a function of contact time was measured by examining the intensity of its characteristic absorption peak at 290 nm in UV–Vis spectra. The relative amount of ^99^TcO_4_^−^ removed was ~92% within the first 30 s and increased to >95% after 5 min. Liquid scintillation counting (LSC) measurements further verified that SCU-103 exhibits extremely rapid kinetics with an equilibrium time of ~5 min (Fig. 3b, Supplementary Table 1). The rapid exchange kinetics may derive from the nature of the layered structure, which is beneficial for the rapid delivery of anions. Moreover, high positive charge density and hydrophobicity also increase the affinity of the 2D layers for ^99^TcO_4_^−^ anions. Note that SCU-103 exhibits very high anion-exchange efficiency in contrast to typical commercial resins (A532E and A530E)^16,18^ and other anion exchangers designed for the removal of anionic contaminants including NDTB-1^13,14^, SLUG-21^39^, UiO-66-NH_3_^+^^48^, comparable to those of SCU-100^16^, SCU-101^42^, and SCU-102^45^. The ultrafast sorption kinetics has great application significance and unique advantages as the short contact time between sorbents and radioactive waste solution would effectively reduce the risk of nuclear leakage and lower the damage of sorbents induced by radiation and hydrolysis.

Considering the high total activity of ^99^Tc sample needed for the anion-exchange isotherm experiment, ReO_4_^−^ was used as a nonradioactive surrogate for ^99^TcO_4_^−^ owing to its similar charge density. In addition, the exchange kinetics for ReO_4_^−^ under the same conditions are almost identical to ^99^TcO_4_^−^ (Fig. 3b, Supplementary Figure 5a and Table 2). Further analysis indicates that the kinetic exchange data for ReO_4_^−^ was fit well with the pseudo-second-order kinetic model as the plot of *t*/*q*_*t*_ *vs t* exhibits a perfect linear relationship with a high correlation coefficient *R*^2^ (> 0.99) (Supplementary Figure 5b), indicating that the ReO_4_^−^/^99^TcO_4_^−^ removal by SCU-103 involves chemical adsorption. The exchange of nitrate anions in SCU-103 by ReO_4_^−^ anions was also confirmed by energy-dispersive spectroscopy (EDS) (Supplementary Figure 6). Elemental distribution mapping of the exchanged products exhibited the presence of a significant amount of captured Re and its homogeneous distributions in the sample (Supplementary Figure 7). In addition, the new peak at 905 cm^−1^ and the decreased intensity of 1332 cm^−1^ in the Fourier-transform infrared spectroscopy (FT-IR) spectra of the ReO_4_^−^ exchanged SCU-103 material (Supplementary Figure 8) confirm the anion-exchange process.

### Sorption isotherm analysis

To evaluate the ReO_4_^−^ uptake capacity of SCU-103, adsorption isotherm experiments at room temperature were executed with the initial concentration of Re ranging from 5 to 400 ppm. As depicted in Fig. 3c, the sorption isotherm curves plotted by the equilibrium concentration against the ion-exchange capacity *q* (mg/g) are fit well with the Langmuir model and the maximum anion-exchange capacity (*q*_m_) was calculated to be 318 ± 8 mg/g (Supplementary Tables 3 and 4). This greatly exceeds the capacity of Yb_3_O(OH)_6_Cl (48.6 mg/g)^49^, NDTB-1 (49.4 mg/g)^49^, UiO-66-NH_3_^+^ (159 mg/g)^48^, NU-1000 (210 mg/g)^47^, SCP-IHEP-1 (211 mg/g)^46^, SCU-101 (247 mg/g)^42^, and SCU-102 (291 mg/g)^45^, but is lower than those of SCU-CPN-1 (999 mg/g)^21^, SCU-100 (541 mg/g)^16^, SLUG-21 (602 mg/g)^39^, PAF-1-F (420 mg/g)^59^.

### Selectivity

For high-level nuclear waste streams, there is a large excess of competing anions, such as NO_3_^−^, SO_4_^2−^, CO_3_^2−^, Cl^−^, and so on, which generally have large detrimental effects on the selective capture of ^99^TcO_4_^−^. Therefore, we investigated the ReO_4_^−^ ion exchange of SCU-103 in the presence of one equivalent of competing anions with different charge numbers, including NO_3_^−^, SO_4_^2−^, CO_3_^2−^, PO_4_^3−^, Cl^−^, ClO_4_^−^. Notably, the removal in all cases could achieve 98% (Fig. 3d) with the *K*_d_ values over 10^5^ mL/g (Supplementary Table 5), indicating the strong affinity and very high selectivity for ReO_4_^−^ by SCU-103 against various competing anions. Generally, anions with higher charge densities such as SO_4_^2−^, CO_3_^2−^, PO_4_^3−^ act as strong competitors for ReO_4_^−^ capture owing to the stronger host–guest electrostatic interactions. For instance, the removal percentages of ReO_4_^−^ for UiO-66-NH_3_^+^ are 15%, 50%, and 20% in the presence of PO_4_^3−^, SO_4_^2−^, and ClO_4_^−^, respectively;^48^ for PAF-1-F, only 21 and 19% of the original concentration could be captured with the presence of PO_4_^3−^ and SO_4_^2−^^59^. Although in the present case, SCU-103 still retains a strong preference toward ReO_4_^−^ despite the presence of PO_4_^3−^ and SO_4_^2−^. For certain types of nuclear waste streams such as Hanford and SRS waste, the competing effects of a huge excess of NO_3_^−^ and SO_4_^2−^ should be taken into consideration when examining ^99^TcO_4_^−^ removal. Therefore, the ReO_4_^−^ uptake selectivity was further checked in the presence of different equivalents of NO_3_^−^ and SO_4_^2−^ anions. As depicted in Fig. 3e, the removal percentages remain higher than 97% for the molar ratios of NO_3_^−^ to ReO_4_^−^ ranging from 1:1 to 20:1 and *K*_d_ values higher than 7.96 × 10^3^ mL/g can still be achieved (Supplementary Table 6). Even at a ratio of 100:1, >88% of ReO_4_^−^ ions could be sequestered, comparable to that of SCU-102^45^. Impressively, when SO_4_^2−^ is present in 6000 fold excess, SCU-103 can still retain high relative amounts of ReO_4_^−^ removal (82%, Fig. 3f) and a high *K*_d_ value of 4.66 × 10^3^ mL/g (Supplementary Table 7). These important results indicate that SCU-103 has high selectivity and strong affinity for ReO_4_^−^/^99^TcO_4_^−^ even in the presence of a large excess of NO_3_^−^ or SO_4_^2−^. This remarkable characteristic makes it an extremely viable candidate for selective removal of ReO_4_^−^/^99^TcO_4_^−^ from waste solutions with high ionic strengths.

### pH effect study

**Fig. 4:**
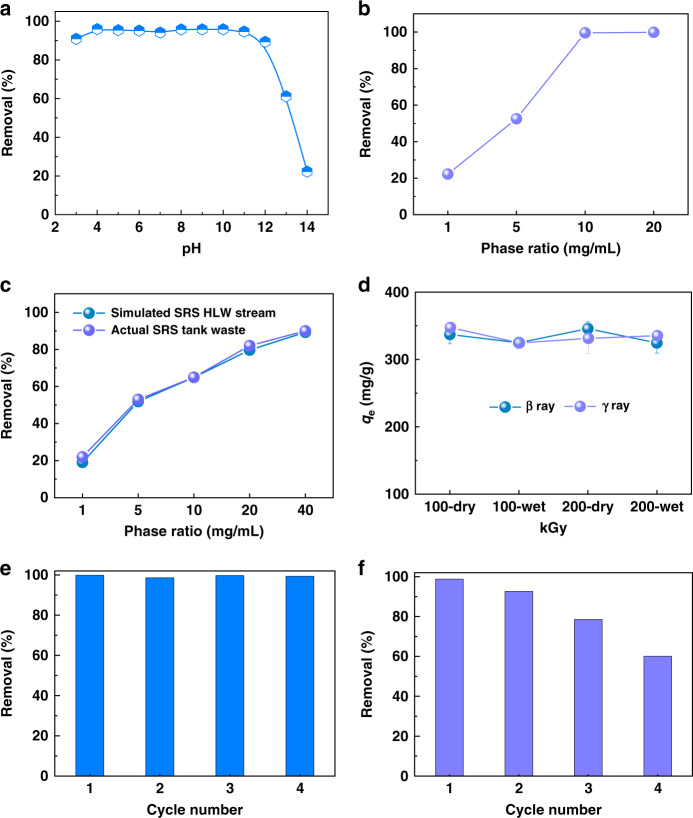
Hydrolytic stability, radiation resistance, and reusability of SCU-103. **a** Effect of pH on the removal of ReO_4_^−^ by SCU-103 (initial concentration of Re: ~200 ppm; solid–liquid ratio of 1 mg/mL). **b** Removal of ReO_4_^−^ by SCU-103 with various solid–liquid ratios in 1 M NaOH solution containing ~200 ppm of Re. **c** ^99^TcO_4_^−^ removal by SCU-103 with various solid–liquid ratios in simulated SRS HLW stream and actual SRS tank waste. **d** ReO_4_^−^ sorption capacities of SCU-103 after different doses of β and γ radiation (initial concentration of Re: ~400 ppm). Error bars represent S.D. *n* = 3 independent experiments. **e** Reusability of SCU-103 for removing ReO_4_^−^ at pH 7 with initial concentration of Re ~28 ppm. **f** Reusability of SCU-103 for removing ReO_4_^−^ in 1 M NaOH containing ~200 ppm of Re.

The removal of ^99^TcO_4_^−^ anions under extreme conditions, such as in acidic and alkaline conditions is highly desirable. The uptake capability of SCU-103 under different pH values were determined in solutions containing 200 ppm of ReO_4_^−^ at a solid–liquid ratio of 1 mg/mL. As shown in Fig. 4a, the removal percentage remains at a high level (>90%) within a wide pH range of 3–12. Even in 0.1 M and 1 M NaOH (defined as pH 13 and pH 14 for convenience), SCU-103 can still afford ReO_4_^−^ removal efficiency of 61% and 22%, respectively. (Supplementary Table 8). Impressively, the removal efficiencies in 1 M NaOH solution can increase to >99% at solid–liquid ratios of 10 mg/mL and above (Fig. 4b, (Supplementary Table 9). Such a ^99^TcO_4_^−^/ReO_4_^−^ removal capability from a highly basic solution is reported for the first time, indicating that SCU-103 is a feasible material for ^99^Tc separation in alkaline nuclear waste inventory.

### ^99^TcO_4_^−^ removal from SRS HLW stream

Encouraged by the results above, we then evaluated ^99^TcO_4_^−^ removal capacity by SCU-103 in a simulated SRS HLW waste sample. In contrast to extremely acidic nuclear waste generated in used nuclear fuel reprocessing, the nuclear inventory at SRS is highly alkaline, containing a sufficient excess of OH^−^ (1.3 M), NO_3_^−^ (2.6 M), NO_2_^−^ (0.1 M), SO_4_^2−^ (0.5 M), and CO_3_^2−^ (0.03 M), in addition to 8.0 × 10^−5^ M TcO_4_^−^ (Supplementary Table 10). Up to now, no material has been demonstrated to be functional with sufficient stability under these conditions. More impressively, the removal of ^99^TcO_4_^−^ from the simulated HLW by SCU-103 is 52% at a phase ratio of 5 mg/mL and ~90% of ^99^TcO_4_^−^ could be extracted at a solid–liquid ratio of 40 mg/mL (Fig. 4c, Supplementary Table 11). A series of batch contact tests were also performed utilizing a sample of actual SRS tank waste with a sufficient excess of OH^−^ (1.88 M), NO_3_^−^ (1.82 M), NO_2_^−^ (0.489 M), SO_4_^2−^ (0.04 M), and CO_3_^2−^ (0.24 M) (Supplementary Table 12)^60^. The actual waste sample also contained significant quantities of various radionuclides including ^137^Cs, ^90^Sr, ^238^Pu, and ^99^Tc, yielding a total gamma activity of 6.3E + 05 pCi/mL, a total beta activity of 8.8E + 05 pCi/mL, and a total alpha activity of 2.1E + 04 pCi/mL. To our best knowledge, this is the first time when an advanced porous material is tested under the real scenario where high alkalinity and ionic strength as well as strong radiation are combined together. As can be seen from the data, the percent removal of ^99^TcO_4_^−^ increases as the phase ratio increases. At the highest phase ratio (40 mg/mL) tested, 90% of the ^99^TcO_4_^−^ was removed from solution within 3 hrs (Fig. 4c, Supplementary Table 13). The batch contact test results utilizing actual SRS tank waste and simulated tank waste are remarkably consistent, indicating that SCU-103 can remove ^99^TcO_4_^−^ efficiently under such conditions of high ionic strengths and high radiation.

### Reusability

We also assessed the reusability of SCU-103 using a neutral solution containing 30 ppm of ReO_4_^−^. The ReO_4_^−^-exchanged SCU-103 materials could be conveniently eluted by applying 1 M NaNO_3_ solutions and more than 98% ReO_4_^−^ could be dissociated back to the solutions. Even after four cycles of sorption/desorption, the removal efficiency of SCU-103 is not affected (Fig. 4e, Supplementary Table 14). The FT-IR analysis also confirms that the loaded SCU-103 material can transform back to the original material after eluting (Supplementary Figure 8). More impressively, SCU-103 also retains good regeneration properties after removing ReO_4_^−^ in 1 M NaOH at a solid–liquid ratio of 10 mg/mL. During the first two sorption/desorption cycles, the removal percentage remained as high as >90% (Fig. 4f, Supplementary Table 15). This high elution efficiency, base stability and easy separation from the treated medium highlight the great potential application for extraction of ^99^TcO_4_^−^ from nuclear waste.

### Sorption mechanism

**Fig. 5:**
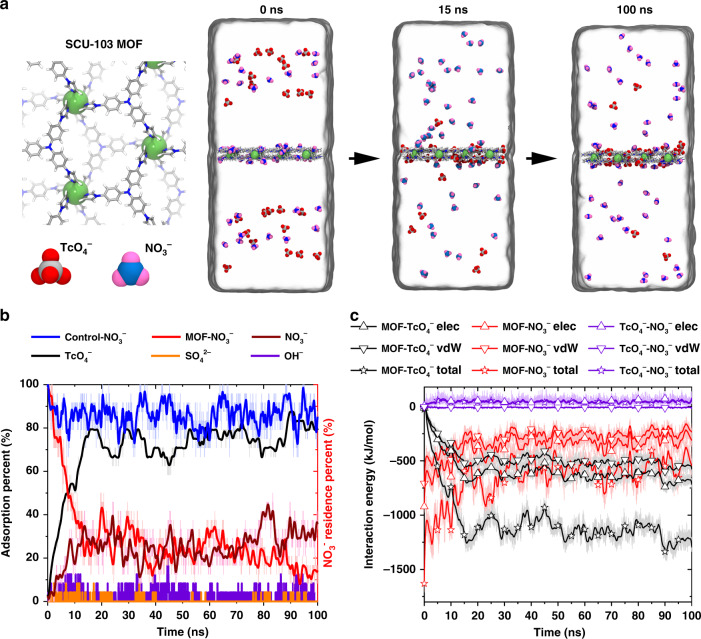
All-atom molecular dynamics simulations for the selective sorption of ^99^TcO_4_^−^ into SCU-103 against various competing anions (including NO_3_^−^, SO_4_^2−^, and OH^−^). **a** Local structure of SCU-103, TcO_4_^−^, and NO_3_^−^ (left panel). Some snapshots at critical time points to show the anion-exchanging process of TcO_4_^−^ over NO_3_^−^ anions (right panel). **b** Adsorption ratios of TcO_4_^−^ (black), NO_3_^−^ (red), SO_4_^2−^ (orange), and OH^−^ (purple) that originally diffused in bulk water, and the residence ratio of NO_3_^−^ originally located in SCU-103 without any competing anions (navy blue curve). **c** Time evolution of the non-bonded interaction energies (including electrostatic part, van der Waals part and their sum) of MOF-TcO_4_^−^, MOF-NO_3_^−^, and TcO_4_^−^-NO_3_^−^.

We studied the selective sorption behavior of ^99^TcO_4_^−^ into SCU-103 against various competing anions (including NO_3_^−^, SO_4_^2−^, and OH^−^) and elucidated the underlying molecular mechanism using all-atom molecular dynamics (MD) simulations. As shown in Fig. 5a, b, after immersing the solid SCU-103 into the aqueous solution that initially contains various anions (i.e., ^99^TcO_4_^−^, NO_3_^−^, SO_4_^2−^, OH^−^, and NO_3_^−^), ~80% of ^99^TcO_4_^−^ in aqueous solution is sorbed into the interior of SCU-103 within a very short period of time (15 ns), whereas 80% of NO_3_^−^ originally residing in SCU-103 is released to the bulk water. Impressively, we observed almost no SO_4_^2−^ or OH^−^ anions in the aqueous solution being sorbed into SCU-103 (<3%), which may also account for its alkaline stability (unfavorable binding free energies for SO_4_^2−^ (21.1 kJ/mol) and OH^−^ (16.6 kJ/mol), Supplementary Figure 9). After ~15 ns, the sorption ratios for each type of anions remained constant, suggesting these results from our simulation converge well (Fig. 5b, and also see the Supplementary Movie 1 in Supplementary for a more intuitive demonstration). In another control simulation in pure water (without any competing anions in the external solution environment), ~90% of the residual NO_3_^−^ anions are reserved in SCU-103 (Fig. 5b, navy blue curve). These results suggest that SCU-103 exhibits a remarkable sorption selectivity towards ^99^TcO_4_^−^ over other competing anions, and the uptake of ^99^TcO_4_^−^ is an anion-exchange process. In addition, we also compared the SCU-103-binding site for the sole NO_3_^−^/^99^TcO_4_^−^ anion pair without any other competing anions (for a better sampling). Clearly, without any interference from other types of competing anions, the preferential binding sites for NO_3_^−^ and ^99^TcO_4_^−^ are partially similar (Supplementary Figure 10). For instance, a majority of NO_3_^−^/^99^TcO_4_^−^ anions prefer being located near the imidazole rings in the cavity of SCU-103 surface and on the top of the imidazole ring above the surface (Supplementary Figure 10). This is reasonable, as these regions carry the highest positive electrostatic potential (Supplementary Figure 11).

To further probe the driving force for this vigorous anion-exchange (NO_3_^−^ replaced by ^99^TcO_4_^−^) process, direct interaction energies between ^99^TcO_4_^−^/NO_3_^−^ and SCU-103, and between ^99^TcO_4_^−^ and NO_3_^−^ as a function of simulation time were computed and analyzed, and they were also decomposed into the contributions from the van der Waals (vdW) and electrostatic (elec) interactions to illustrate the major driving force. During the main ^99^TcO_4_^−^ sorption stage (*t* < 15 ns), the direct interaction between ^99^TcO_4_^−^ and SCU-103 drives the sorption of ^99^TcO_4_^−^, as witnessed by the sharply lowered (more favorable) interaction energy from 0 to ~−1250 kJ/mol (black hollow star in Fig. 5c). The contribution from the electrostatic interaction (~700 kJ/mol) (black hollow triangle in Fig. 5c) is slightly larger than that from the vdW interaction (~550 kJ/mol) (black hollow inverted triangle in Fig. 5c). While, during the same period, the direct interaction energy between NO_3_^−^ and SCU-103 is increased by 1000 kJ/mol (from ~−1600 kJ/mol to ~−600 kJ/mol; red hollow star in Fig. 5c) (less favorable), with the electrostatic interaction contributing ~600 kJ/mol (red hollow triangle in Fig. 5c) and the vdW interaction contributing the other ~400 kJ/mol (red hollow inverted triangle in Fig. 5c). Again, the electrostatic interaction contributes slightly more than van der Waals interaction. Meanwhile, owing to direct electrostatic repulsion, the interaction energy between ^99^TcO_4_^−^ and NO_3_^−^ increased from 0 to 50 kJ/mol (repulsive) (purple hollow star in Fig. 5c). These results suggest that direct “^99^TcO_4_^−^ anions−SCU-103” non-bonded interactions, with electrostatic interaction contributing slightly more than vdW interaction, has a critical role in the ^99^TcO_4_^−^ uptake process.

**Fig. 6:**
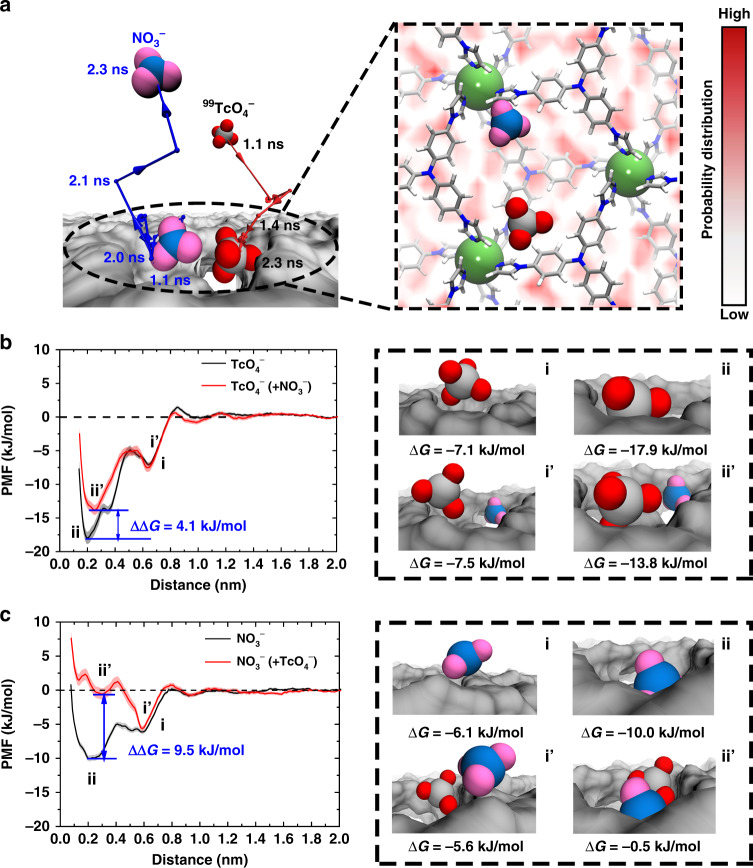
Binding free energies of ^99^TcO_4_^−^ and NO_3_^−^ to their most energetically favorable sites in SCU-103. **a** A representative anion-exchange process of NO_3_^−^ by TcO_4_^−^ (left). The red and blue lines with an arrow represent the center of mass motion trajectory of TcO_4_^−^ and NO_3_^−^ from 1.1 ns to 2.3 ns. The most important crucial intermediate state during the anion-exchange process is shown when TcO_4_^−^ binds to its final binding site, while NO_3_^−^ dwells at its original binding site (right). The color gradient of the background demonstrates the binding probability distribution of NO_3_^−^ to SCU-103, and ranges from lowest probability (0, white) to highest probability (0.110, red). **b** and **c** Binding free energies of TcO_4_^−^/NO_3_^−^ alone to its final binding site (black curves), and the binding free energies of the two anions at the crucial intermediate state (red curves). Snapshots highlighted by the black dashed lines represent the typical binding configurations of TcO_4_^−^/NO_3_^−^ anions to SCU-103 corresponding to the energy minima in the free energy curves. The shadows in **b** and **c** represent the predictive error of the binding free energy profiles.

A representative anion-exchange event was then analyzed, with its most important intermediate state in the process revealed. As shown in Fig. 6a and Supplementary Movie 2 in Supplementary, initially (*t* < 1.1 ns), a NO_3_^−^ anion resides very close to an imidazole ring in the interior binding cavity of SCU-103. From ~1.1 ns to ~1.4 ns, a ^99^TcO_4_^−^ anion gradually approaches the same binding cavity of the NO_3_^−^, and transiently stays on the top of the imidazole ring. From 1.4 ns to ~ 2.3 ns, the ^99^TcO_4_^−^ anion enters the cavity, and is stably anchored to the site close to another adjacent imidazole ring. It is noteworthy that, during the main intruding stage of ^99^TcO_4_^−^ into the binding cavity (1.1 ns~2.0 ns), NO_3_^−^ oscillates around its original binding site. However, after a very short ~0.1 ns (i.e., *t* = ~2.1 ns), NO_3_^−^ is expelled out of its original binding site, and rapidly (from 2.1 ns to 2.3 ns) diffuses into the bulk water, completing the anion-exchange process. Throughout this entire exchange dynamic, clearly, the state when ^99^TcO_4_^−^ is bound to its final binding site while NO_3_^−^ dwells at its original binding site is the most crucial intermediate state.

The binding free energies (potential of mean force, PMF) of ^99^TcO_4_^−^/ NO_3_^−^ was then calculated for this most important intermediate state. We first investigated the changes in the binding free energy of the two anions to SCU-103 at this critical state, and then compared the values obtained from each of the two anions solely (in the absence of other competing anions) bound to its binding site. The black PMF curves in Fig. 6b, c demonstrate that during the pathway of NO_3_^−^/^99^TcO_4_^−^ alone binding to its final binding site, there are two prominent binding free energy minima. In the case of ^99^TcO_4_^−^ (Fig. 6b, black curve), the global minimum is located at the distance of ~0.20 nm (very close to an imidazole ring within the binding cavity), with a Δ*G* of −17.9 kJ/mol. Another local minimum is located at the distance of 0.64 nm (on the top of an imidazole ring above SCU-103), with a Δ*G* of −7.1 kJ/mol. The two minima are separated by a free energy barrier ~2.2 kJ/mol (~1 k_B_T at room temperature), which can be easily overcome by thermal fluctuation. Similarly, in the case of NO_3_^−^ (Fig. 6c, black curve), the global minimum is located at the distance of ~0.21 nm (very near another imidazole ring within the binding cavity), with a Δ*G* of −10.0 kJ/mol, whereas the other local minimum is situated at the distance of ~0.59 nm (on the top of another imidazole ring above the binding cavity), with a Δ*G* of −6.1 kJ/mol. These two minima are also separated by a moderate energy barrier of ~2.1 kJ/mol. This indicates that both anions are energetically favorable to bind to SCU-103. Meanwhile, ^99^TcO_4_^−^ shows a notably stronger binding affinity than NO_3_^−^, which indicated that the anion-exchange process is thermodynamically feasible. More specifically, the binding free energies for ^99^TcO_4_^−^ and NO_3_^−^ at their most energetically favorable binding sites in SCU-103 are −17.9 kJ/mol and −10.0 kJ/mol, respectively. At the crucial intermediate state, the binding of ^99^TcO_4_^−^ can significantly diminish the global binding free energy minimum of NO_3_^−^ at the distance of 0.21 nm, and meanwhile, the binding free energy of NO_3_^−^ at the position is remarkably increased by a ΔΔ*G* = 9.5 kJ/mol, which eventually reaches −0.5 kJ/mol (nearly comparable to the bulk value) (Fig. 6c, red curve). The data imply that the electrostatic repulsion introduced by ^99^TcO_4_^−^ can completely offset the binding affinity between NO_3_^−^ and SCU-103, leading to the diffusion of NO_3_^−^ from the interior of SCU-103 into the bulk water. At the transition state, the binding affinity of ^99^TcO_4_^−^ can also be weakened by NO_3_^−^, e.g., the binding free energy of ^99^TcO_4_^−^ at the distance of 0.20 nm increases from −17.9 kJ/mol to −13.8 kJ/mol (Fig. 6b, red curve). However, this value is still the global minimum throughout the full ^99^TcO_4_^−^ adsorption PMF curve (Fig. 6b, red curve), suggesting the moment when ^99^TcO_4_^−^ is bound close to the imidazole ring within the cavity still represents the most energetically favorable binding state during the whole process.

## Discussion

The foregoing results demonstrate a rare example of an alkaline-stable cationic MOF with excellent ^99^TcO_4_^−^ capture capabilities in actual SRS HLW streams, potentially overcoming the long-term challenge in legacy waste partitioning. The unique concave–convex layers containing sterically crowded metal centers in SCU-103 provide suitable recognition sites for the selective incorporation of ^99^TcO_4_^−^ and sufficient resistance to H_2_O and OH^−^ attack under basic conditions, which is further supported by the combined density functional theory calculation and molecular dynamics simulation. This work not only reports the first example, to the best of our knowledge, of advanced porous materials showing application in a real scenario that combines the extreme conditions of high alkalinity, large excess of competing anions, and strong radiation, but also provides a new design philosophy on solid sorbent materials for the remediation of critical environmental pollutants in the future.

## Methods

Caution! ^99^Tc is a β-emitter (*t*_1/2_ = 2.13 × 10^5^ a) and possesses significant health risks when inhaled or ingested. Standard precautions and procedures for handling radioactive materials should be followed, and all ^99^Tc studies were conducted in licensed laboratories dedicated to radiological investigations (i.e., Soochow University and Savannah River National Laboratory).

### Reagents and materials

Tris[4-(1H-imidazol-1-yl)-phenyl]amine (tipa) was purchased from Jinan Camolai Trading Co., LTD. Ni(NO_3_)_2_·6H_2_O, HNO_3_ (65~68%), NaOH (AR), and DMF were provided by Sinopharm Chemical Reagent Co., Ltd. NaReO_4_ (99%) was purchased from Alfa Aesar (China) chemical Co., Ltd. All the chemicals were used without further purification. ^99^TcO_4_^−^ stock solutions were prepared by dissolving desired amounts of NH_4_^99^TcO_4_ (99%) solid in deionized water.

### Physical property measurements

PXRD patterns were measured from 5 to 50° on a Bruker D8 Advance diffractometer by using Cu*Kα* radiation (*λ* = 1.54056 Å) with a step of 0.02 and a Lynxeye one-dimensional detector. The FT-IR were determined in the range of 4000 to 400 cm^−1^ on a Thermo Nicolet iS50 spectrometer. EDS and the elemental distribution maps were recorded on a JEOL JSM-6700F scanning electron microscope (SEM). Thermogravimetric analysis was performed on a NETZSCHSTA 449F3 instrument with a constant rate of 10 K/min from 30 to 900 °C under a N_2_ flow. Inductively coupled plasma-optical emission spectroscopy (ICP-OES) measurement was performed on a Thermo Fisher Scientific iCAP 7200 to determine the concentrations of ReO_4_^−^. Absorption spectra of ^99^TcO_4_^−^ were carried out on a Cary 6000i spectrophotometer (Agilent Inc.) from 200 to 400 nm with an interval of 0.1 nm. The concentrations of ^99^TcO_4_^−^ in kinetics studies and exchange experiments with simulated SRS HLW stream were evaluated by ^99^Tc activity utilizing an ultralow level Tri-Carb 2910TR LSC. In the batch exchange experiments with actual SRS tank waste, ^99^Tc activity in the samples was also measured by LSC. The ^99^TcO_4_^−^ was separated from the tank waste matrix using Eichrom TEVA resin. Samples were also spiked with ^99m^Tc prior to separation to trace the recovery of technetium throughout the radiochemical separation. The ^99m^Tc activities were measured by gamma spectroscopy. The separation recovery was then applied to the ^99^Tc activities measured by LSC to provide results accounting for each sample’s individual recovery.

### Synthesis of SCU-103

A mixture of Ni(NO_3_)_2_·6H_2_O (0.058 g; 0.20 mmol), tris(4-(imidazol-1-yl)pheny)amine (tipa) (0.086 g; 0.20 mmol), DMF (3 mL) and deionized water (1.5 mL) was loaded into a 20 mL Teflon-lined stainless steel autoclave. The container was closed, heated at 140 °C for 3 days, and then cooled to room temperature at a cooling rate of 5 °C h^−1^. A pure phase of brown hexagonal block-like crystals was collected after washing the product with ethanol three times (Yield: 0.065 g; 60.8% based on tipa).

### Single-crystal structure determination

A single crystal for single-crystal X-ray diffraction was carefully selected under an optical microscope. Data were collected on a Bruker D8-Venture diffractometer with a Turbo X-ray Source (Mo*Kα* radiation (*λ* = 0.71073 Å) at 296 K. The data frames were collected using the program APEX 3 and processed using the program SAINT routine in APEX 3. The structures were solved by direct methods and refined by full-matrix least-squares on *F*^2^ using the SHELX-2016 program package^61^. All non-hydrogen atoms were refined anisotropically and the hydrogen atoms bonded to C atoms were located at geometrically calculated positions. The disordered NO_3_^−^ anions and solvent molecules have been squeezed. The “squeeze” was performed by *PLATON* using INS or CIF file^62^. The unit cell was found to contain one void of 2683 Å^3^, holding 607 electrons, which are contributed by disordered NO_3_^−^ anions and solvent molecules (H_2_O and DMF). According to the thermogravimetric analysis, the disordered solvents in one formula unit could be approximately identified as four H_2_O and two DMF molecules (Supplementary Figure 13). Therefore, two disordered NO_3_^−^ anions, four H_2_O, and two DMF molecules contribute 184 electrons in one formula unit. Thus, the voids of one unit cell contain 552 electrons, which are close to the 607 electrons calculated by the *SQUEEZE/PLATON* procedure. The quality of the crystals was not good and the diffraction intensity was very weak, especially at high angles, thus resulting in poor crystal data with high *R*_int_ value. We have tried our best to improve the quality of the data, including re-growing crystals, changing collection methods, but unfortunately failed. CCDC 1985724 contains the supplementary crystallographic data for this paper. These data can be obtained free of charge from the Cambridge Crystallographic Data Centre.

### Batch experiments

All the experiments were performed at room temperature (~25 °C) using the batch sorption method with the solid/liquid ratio of 1 g L^−1^. Typically, 5 mg of SCU-103 was added into 5 mL of aqueous solution of ReO_4_^−^/^99^TcO_4_^−^ with different concentrations. The mixture was kept under magnetic stirring for a desired contact time. The concentrations of ReO_4_^−^ in the clear supernatant, which was filtered by a 0.22-μm nylon membrane filter on a 2-mL syringe and diluted with aqueous solution of HNO_3_ (mass fraction, 2%) to meet the concentration range of the test instrument, were determined by ICP-OES. Standard ReO_4_^−^ solutions with the concentrations of 0, 1, 2.5, 5, 10 ppm were used to build standard curves with the linear correlation coefficient >0.999. After anion exchange, the polycrystalline materials were washed with deionized water several times and air-dried, and then characterized by FT-IR spectroscopy, PXRD, and SEM-EDS.

### Exchange kinetics studies of SCU-103

The kinetic studies of ^99^TcO_4_^−^ ion exchange by SCU-103 were done by performing the ion-exchange experiments with various contact times. In all, 20 mg of SCU-103 which had been ground into polycrystalline powder was weighed into a 20 mL solution containing 14 ppm of ^99^TcO_4_^−^, and the mixtures were kept under magnetic stirring for the desired contact times. The concentration of ^99^TcO_4_^−^ as a function of contact time was probed by the UV–vis spectra using the absorption peak at 290 nm. In addition, ^99^Tc activity was also determined by LSC. The uptake kinetics experiment was then repeated using 28 ppm of ReO_4_^−^ as the surrogate for ^99^TcO_4_^−^ for comparison. The kinetic data for the ReO_4_^−^ removal were further analyzed with pseudo-second order kinetics, which is used to determine that the rate-determining step is governed by chemical adsorption. The equation is defined as:

1 $$\frac{t}{{q_t}}{\mathrm{ = }}\frac{1}{{k_2q_e^2}}{\mathrm{ + }}\frac{t}{{q_e}}$$

*t* refers to the contact time (min); *q*_*t*_ and *q*_e_ are the amounts of the Re that absorbed by per unit weight of sorbents (mg g^−1^) at time *t* and equilibrium time, respectively. *k*_2_ is the pseudo-second-order rate constant of adsorption (g mg^−1^ min^−1^). *q*_e_ and *k*_2_ can be calculated from the slope and intercept of the line plots of *t*/*q*_*t*_ *vs t*.

### Sorption isotherm experiments

The sorption isotherm experiments of SCU-103 towards ReO_4_^−^ were conducted by varying the initial ReO_4_^−^ concentrations from 5 to 400 ppm. In all, 5 mg of sorbent was placed into 5 mL of aqueous solution containing a certain concentration of ReO_4_^−^. The resulting mixture was stirred for 12 h to ensure equilibrium was reached and then separated using a 0.22-μm nylon membrane filter. The concentrations of ReO_4_^−^ in the clear supernatant were analyzed by ICP-OES. The experimental sorption isotherm curves, derived from the equilibrium concentration *C*_e_ (ppm) plotted against the corresponding anion-exchange capacity *q* (mg/g), were fitted by Langmuir and Freundlich isotherm equations.

In the Langmuir isotherm model, it is hypothesized that the sorption is monolayer sorption, all the adsorption sites are equivalent and the energy of adsorption is constant. It is also presumed that the absorbed ions on adjacent sites are independent and there is no interaction between them. It can be described by Eq. (2).

2 $$q = q_m\frac{{K_LC_e}}{{{\mathrm{1 + }}K_LC_e}}$$

where *q* (mg g^−1^) is the exchanged amount of the ions at the equilibrium concentration *C*_e_ (mg L^−1^), *q*_m_ refers to the maximum exchange capacity of the exchangers; *K*_L_ (L/mg) is the Langmuir constant indirectly related to the free energy of the exchange, which characterizes the affinity between ions and absorbents.

The Freundlich model assume that sorption occurs on the heterogeneous surface, the binding energies differ at adsorption sites, and the binding strengths decrease with the increase of occupied sites. The equation can be expressed by:

3 $$q = K_FC_e^{1/n}$$

where *K*_F_ and *n* are the Freundlich constants related to the sorption capacity and the sorption intensity, respectively. The fitting results of two sorption models are listed in Supplementary Table 4.

### Anion competition studies

The competitive ion-exchange experiments were carried out at the solid/liquid ratio of 1 g L^−1^, room temperature (~25 °C), with the contact time of 12 h. The competing effect of anions including SO_4_^2−^, NO_3_^−^, CO_3_^2−^, PO_4_^3−^, Cl^−^, ClO_4_^−^ were initially performed by loading 0.5 mM Na_2_SO_4_, NaNO_3_, Na_2_CO_3_, NaH_2_PO_4_, NaCl, or NaClO_4_ solutions into 0.5 mM ReO_4_^−^ solution, respectively. The effect of excessive NO_3_^−^ anion was examined by adding 0.15 mM, 0.75 mM, 1.5 mM, 3 mM, or 15 mM NaNO_3_ solutions, respectively, into a 0.15 mM ReO_4_^−^ solution. The ReO_4_^−^ sorption capacity of SCU-103 in the presence of different concentrations of SO_4_^2−^ was further conducted by adding 0.08 mM, 0.80 mM, 8.0 mM, 80 mM, or 480 mM Na_2_SO_4_ solutions into a 0.08 mM ReO_4_^−^ solution, respectively. The concentrations of ReO_4_^−^ after sorption in aqueous solution were determined by ICP-OES.

### pH effect study

The solution pH was adjusted by adding negligible volumes of diluted nitric acid or sodium hydroxide. The effect of pH on ReO_4_^−^ sorption was carried out by varying pH values from 2 to 14.5 mg of SCU-103 was added to 5 mL of aqueous solution containing 200 ppm of ReO_4_^−^. After being shaken at a rate of 100 rpm for 12 h on an oscillator, the resulting mixture was separated with a 0.22-µm nylon membrane filter for ICP-OES analysis.

### Exchange experiments in 1 M NaOH system

The exchange experiments were performed by mixing 1 M NaOH aqueous solutions containing 200 ppm ReO_4_^−^ with SCU-103 crystals to provide solid/liquid ratios of 1, 5, 10, 20 g L^−1^. After being stirred for 12 h, the suspension was separated with a 0.22-µm nylon membrane filter for ICP-OES analysis.

### Exchange experiments with SRS HLW stream

A simulated SRS HLW Stream was prepared according to a reported protocol^22^ and the molar concentration of the anions and molar ratio of each anion to that of ^99^TcO_4_^−^ are provided in Supplementary Table 10. Measured quantities of the simulated SRS HLW Stream were pipetted into sample tubes containing a premeasured quantity of SCU-103 to provide solid/liquid ratios of 1, 5, 10, 20, and 40 g L^−1^. The sample tubes were placed on an oscillator for 3 h of shaking at ambient temperature. The suspension was separated with a 0.22-μm nylon membrane filter and the filtrate was analyzed by liquid scintillation counting to determine the ^99^Tc activity.

A series of batch contact tests were also performed utilizing a sample of actual SRS tank waste with the composition shown in Supplementary Table 11^60^. The ^99^Tc activity of this sample was 4.3E+04 pCi/mL. Other key radionuclides in the sample included ^137^Cs (6.7E+05 pCi/mL), ^90^Sr (2.9E+04 pCi/mL), and ^238^Pu (2.5E+04 pCi/mL). A tank downstream of the cesium removal process was selected in order to provide a sample with a relatively low dose (compared with before Cs removal) for working with in the laboratory. Samples of SCU-103 were added to 15-mL conical bottom polypropylene tubes in amounts ranging from 0.004 to 0.16 g. To each tube was then added 4 mL of the SRS tank waste solution to yield experiments with phase ratios ranging from 1 mg/mL to 40 mg/mL. A tube containing only the SRS tank waste solution (no SCU-103) was also run in parallel as a control sample. The tubes were then mounted on a Thermo Scientific Labquake tube rotator and were tumbled for 3 hrs. At the end of the 3-hour experiment, the tubes were removed from the rotator. The supernatant from each tube was then decanted and filtered through a 0.1-µm polyvinylidene difluoride syringe filter. The filtrate was analyzed for ^99^Tc activity.

### Radiation-resistance measurements

The β-ray was provided by an electron accelerator equipped with an electron beam (10 MeV) and the γ-ray radiation was provided by a ^60^Co radiation source. Dry crystals of SCU-103 or crystals immersed in 0.5 mL of H_2_O were irradiated at two different doses (100 and 200 kGy), respectively. The radiation resistance of SCU-103 was characterized by PXRD measurements and further checked by ReO_4_^−^ uptake capacity experiments using the irradiated samples.

### Exchange reversibility studies

In order to elute the materials, 50 mg of ReO_4_^−^ exchanged products were used in the elution experiments by applying 50 mL of 1 M NaNO_3_ aqueous solution under shaking for 12 h on the oscillator at room temperature (~25 °C). After this treatment, the solid samples were filtered and washed with deionized water, dried, and analyzed with PXRD and FT-IR measurements. The regenerated products were used for ReO_4_^−^ uptake experiments, and the process was repeated for multiple runs.

### Computational method

The dynamic trajectories of the selective sorption of ^99^TcO_4_^−^ into SCU-103 and the corresponding anion-exchange process were performed using GROMACS 5.1.4^63^ applying the OPLS-AA force field (FF)^64^. This FF has been previously used to investigate the hydration properties of ions as well as the interactions between radioactive anions and various porous materials with good compatibility^20,51,65–68^. For a detailed description of the force field parameters and simulation system see the Supporting Information.

## Supplementary information

Supplementary Information

Supplementary Movie 1

Supplementary Movie 2

## Data Availability

The data that support the findings of this paper are available in the paper and supplementary information files. The X-ray crystallographic data for the structure reported in this article has been deposited at the Cambridge Crystallographic Data Centre (CCDC) with the number of CCDC 1985724. These data can be obtained free of charge from The Cambridge Crystallographic Data Centre via www.ccdc.cam.ac.uk/data_request/cif. Any further relevant data are available from the authors upon reasonable request. Source data are provided with this paper.

## Source data

Source Data
