# Supplementary material for: 99TcO4− removal from legacy defense nuclear waste by an alkaline-stable 2D cationic metal organic framework
Source: Nat Commun. 2020 Nov 4;11:5571. doi: 10.1038/s41467-020-19374-9 (PMC7642432; doi:10.1038/s41467-020-19374-9)
Supplement: Supplementary file 1 — Supplementary Information [file 41467_2020_19374_MOESM1_ESM.pdf]

Supporting Information for

$^{99}TcO_4^-$ Removal from Legacy Defense Nuclear Waste by an Alkaline-Stable 2D Cationic Metal-Organic Framework

Nannan Shen[1,†], Zaixing Yang[1,†], Shengtang Liu[1,†], Xing Dai[1], Chengliang Xiao[2*], Kathryn Taylor-Pashow[3], Dien Li[3], Chuang Yang[1], Jie Li[1], Yugang Zhang[1], Mingxing Zhang[1], Ruhong Zhou[1*], Zhifang Chai[1], Shuao Wang[1*]

[1] State Key Laboratory of Radiation Medicine and Protection, School for Radiological and Interdisciplinary Sciences (RAD-X) and Collaborative Innovation Center of Radiation Medicine of Jiangsu Higher Education Institutions, Soochow University, Suzhou 215123, China.

[2] College of Chemical and Biological Engineering, Zhejiang University, Hangzhou 310027, China.

[3] Savannah River National Laboratory, Aiken, SC 29808, United States.

† These three authors contributed equally.

Molecular Dynamics Simulations. Since the exposed surface of SCU-103 is the main place for anions adsorption and exchange, we adopted the single layer SCU-103 membrane as a representative model to investigate the competing adsorption behaviors of various anions. The SCU-103 model used consisted of 12 repeat units (1428 atoms) along the (111) plane of bulk SCU-103 materials. Each unit was made up of one Ni^{2+} with 1/6 occupancy, six-coordinated ligands (tipa) with 1/3 occupancy and two nitrate anions. Moreover, 24 NO_3^- anions resided in the SCU-103 (due to NO_3^- being used as the counter ion during the synthesis of SCU-103). The SCU-103 membrane was placed into the simulated radioactive aqueous solution, which contained 24 $^{99}TcO_4^-$, 24 NO_3^- 24 OH^-, 24 SO_4^{2-}, 24 NH_4^+, 96 Na^+, and 11,118 water molecules, thus giving a total of 35,328 atoms. The simulation box has a size of (5.58 nm × 4.83 nm × 14.00 nm). The SCU-103 membrane was also solvated into a pure water box with a size of (5.58 nm × 4.83 nm × 5.00 nm) containing 3,893 water molecules, which gave a total of 13,107 atoms. This system served as the control system to check the reservation ratio of residual NO_3^- in SCU-103. The partial charges for SCU-103 atoms were obtained from density functional theory (DFT) calculations based on the unit cell of SCU-103 (find more details in the DFT calculation section). The Lennard-Jones (L-J) parameters for the MOF atoms were obtained from all-atom optimized potentials for liquid simulations (OPLSAA) force field (FF).[1] The Lorentz–Berthelot combining rules were used for calculating cross L-J interaction parameters. The parameters of the $^{99}TcO_4^-$, NO_3^-, OH^-, SO_4^{2-}, NH_4^+ and Na^+ were taken from previous simulation studies.[2-5] We revalidated the hydration free energies and diffusion coefficients of $^{99}TcO_4^-$, NO_3^-, OH^-, and SO_4^{2-} and the results showed high agreement with experimental results (Supplementary Table 15), suggesting the reasonable accuracy of the force filed parameters of various anions used here.

All molecular dynamics simulations (MD) simulations were performed with the GROMACS software package (version 5.1.4).[6] VMD software (version 1.9.3) was applied for trajectory visualization and analysis.[7] The SPC/E water model[8] was used for water. In the production run, a trajectory of 100 ns was generated, a time step of 2.0 fs was used, and the data were collected every 10 ps. All production runs were under isothermal-isochoric (NVT) ensemble. The temperature and pressure were maintained at 300 K and 1 bar using a v-rescale thermostat[9] and the Berendsen barostat algorithm.[10] The short-range electrostatic and van der Waals interaction cutoff distance were set to 1.2 nm. The long-range electrostatic interaction was performed using the particle-mesh Ewald (PME) methodc.[11] All solute bonds between heavy atoms and hydrogen atoms were constrained with the LINCS algorithm.[12] Periodic boundary conditions were applied in all directions, and the MOF was frozen throughout the simulation process.

The hydration free energy of $^{99}TcO_4^-$, NO_3^-, OH^-, and SO_4^{2-} was calculated using the thermodynamic integration (TI) method as the following equation:

$$\Delta G = \sum_i^{n_{steps}} \langle \frac{\partial \mathcal{H}}{\partial \lambda} \rangle_\lambda \Delta\lambda \quad (4)$$

where λ was the coupling parameter for defining the mutation of dummy anion to the anion that grew into the water with different Hamiltonian. The angle brackets indicated the Boltzmann-weighted average level. We adopted 46 λ points for coupling non-bonded interaction (λ = 0.00, 0.001, 0.01, 0.05, 0.10, 0.15, 0.20, 0.25, 0.30, 0.35, 0.40, 0.45, 0.50, 0.55, 0.60, 0.65, 0.70, 0.75, 0.80, 0.85, 0.90, 0.95, 0.99, 0.999, 1.00 for turning on the van der Waals interaction, and λ = 0.00, 0.05, 0.10, 0.15, 0.20, 0.25, 0.30, 0.35, 0.40, 0.45, 0.50, 0.55, 0.60, 0.65, 0.70, 0.75, 0.80, 0.85, 0.90, 0.95, 1.00 for turning on the electrostatic interaction, respectively). The soft-core L-J potential[13] was used to avoid the free energy perturbation "endpoint catastrophe". The umbrella-sampling was adopted to investigate the potential of mean force (PMF) associated with moving a single $^{99}TcO_4^-$, NO_3^-, OH^- and SO_4^{2-} toward the SCU-103. The reaction path for the PMF calculation was defined by pulling the target anion from the bulk water to the center of mass of the MOF membrane (from 2.1 nm to 0.2 nm) with a harmonic force of 1000 kJ/mol·nm^2. The reaction coordinate was divided into 20 windows, with each window simulated for 3 ns and thus accumulating a total simulation of 60 ns. The weighted histogram analysis method (WHAM)[14,15] was applied to calculate the free energy. The error bars for PMF were obtained using bootstrap method.[16] All PMF profiles were normalized to zero at a distance of 2.0 nm. To assess the influence in the binding free energy between $^{99}TcO_4^-$ and NO_3^- in the binding sites, the opposite anion was constrained with a harmonic force of 1000 kJ/mol·nm^2 when the PMF calculations were performed for the other unconstrained anion.

Density Functional Theory Calculations. Density functional theory (DFT) calculations were performed in the Dmol3 program[17,18] to optimize the geometric structure of the SCU-103. The unit cell of SCU-103 ($NiC_{54}N_{14}H_{42}$) was used as the computational model. During geometry optimization, the lattice parameters (a = b = c = 12.380 Å, α = β = γ = 81.200°, obtained from the experimental crystal data), the positions of the Ni atoms and the six N atoms coordinated to Ni were frozen. Other atoms were allowed to freely relax. Geometry optimization was performed at PBE/DNP level[19] with the k-point set of 3×3×3. The DFT semi-core pseudo potential (DSPP) was used as the core treatment for the Ni atom. Subsequently, a periodic monolayer slab model ($NiC_{54}N_{14}H_{42}$, a = b = 16.113 Å, α = β = 90°, γ = 120.000°) with more than 30 Å vacuum region was constructed based on the optimized

unit cell to calculated the electrostatic potential (ESP) distributed on the electron density surface (isodensity = 0.002 a.u.). The PBE/DNP level with the k-point of 3×3×1 was used in ESP calculations.

The atomic charges were calculated with the Gaussian 09 program.[20] A fragment model containing the full coordination environments of Ni was constructed based on the optimized unit cell (Supplementary Figure 12). Single point energy was calculated at the B3LYP/6-31G(d) level[21-23] in the liquid phase by using the polarizable continuum models (PCM).[24] Then, the atomic charges fit ESP at points selected according to the CHelpG scheme[25] were calculated. The calculated atomic charges were used in subsequent classical molecular dynamics simulations. The electrostatic potential surface is shown in Supplementary Figure 11.

Figures:

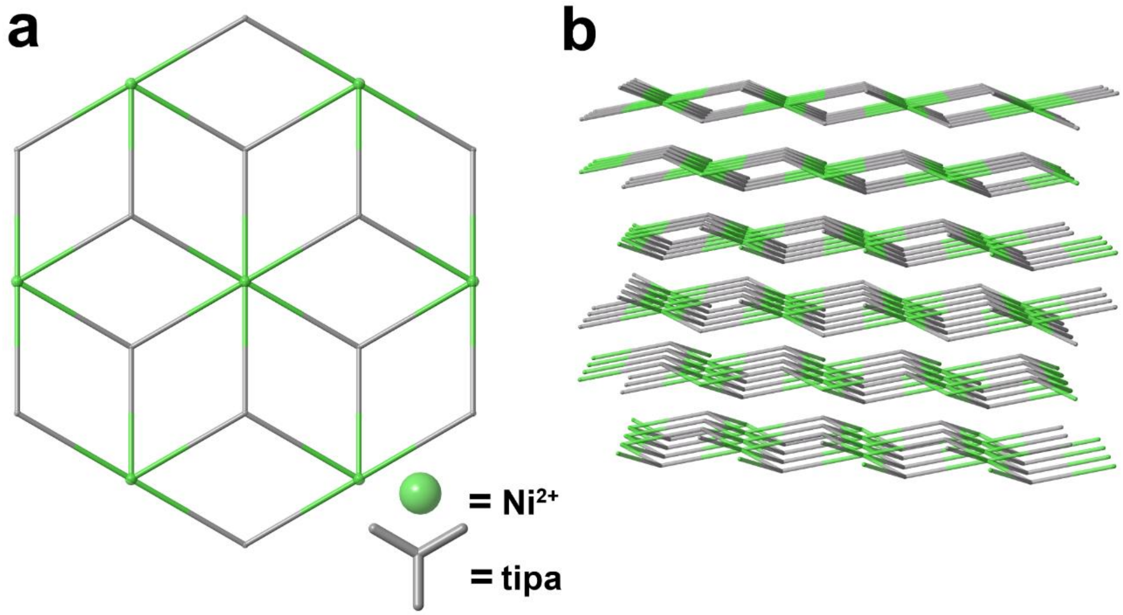

Supplementary Figure 1. Simplified binodal (3,6)-connected *kgd* topology of **SCU-103**.

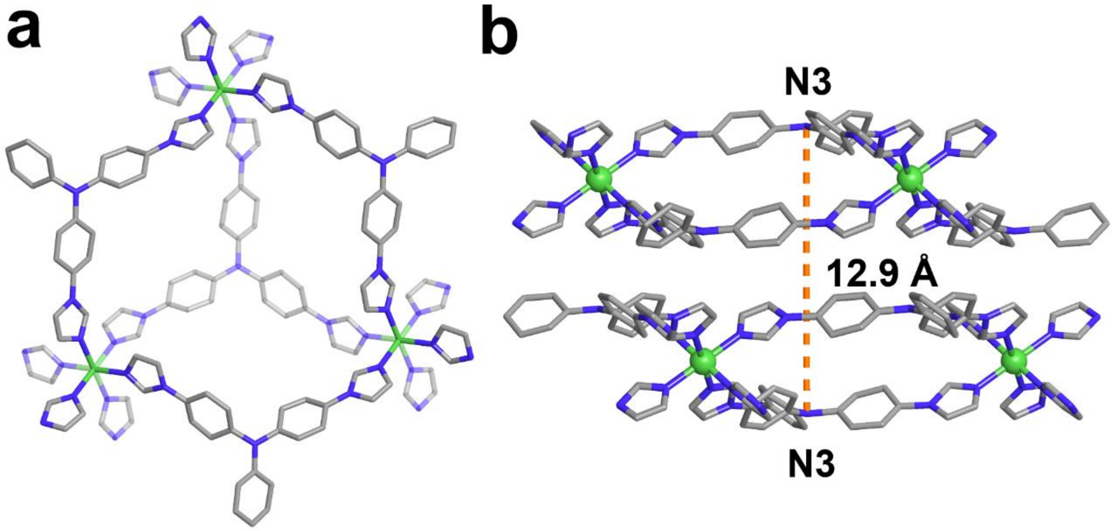

Supplementary Figure 2. (A) Diagram of the bowl-shaped void formed by three Ni^{2+} ions and tipa ligands. (B) N3···N3 distance of coordination environment of Ni^{2+} ion with six ligands.

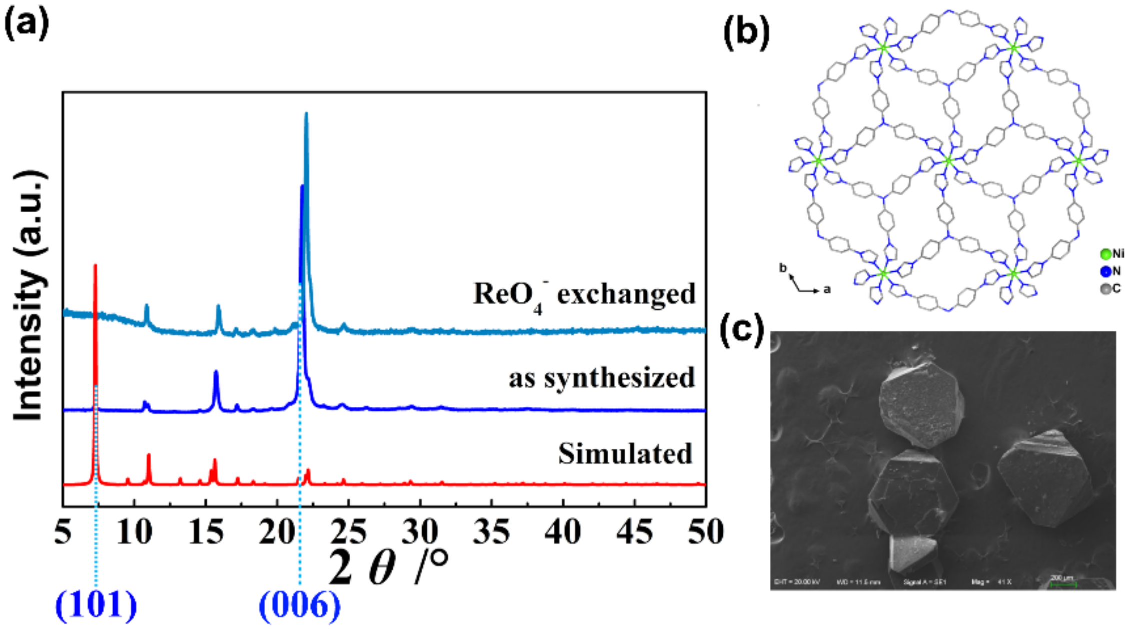

Supplementary Figure 3. (a) PXRD patterns of the simulated SCU-103, as synthesized samples and experimental ReO_4^--exchanged products indicating the experimental patterns exhibit preferred orientation toward (006) facet, which is the orientation of two-dimensional cationic layers (b). (c) Scanning electron microscopy (SEM) images of SCU-103 showing that the as synthesized crystals feature a hexagonal lamellar stacking morphology and a highly exposed facet of (006)/(001), further confirming the presence of preferred orientation.

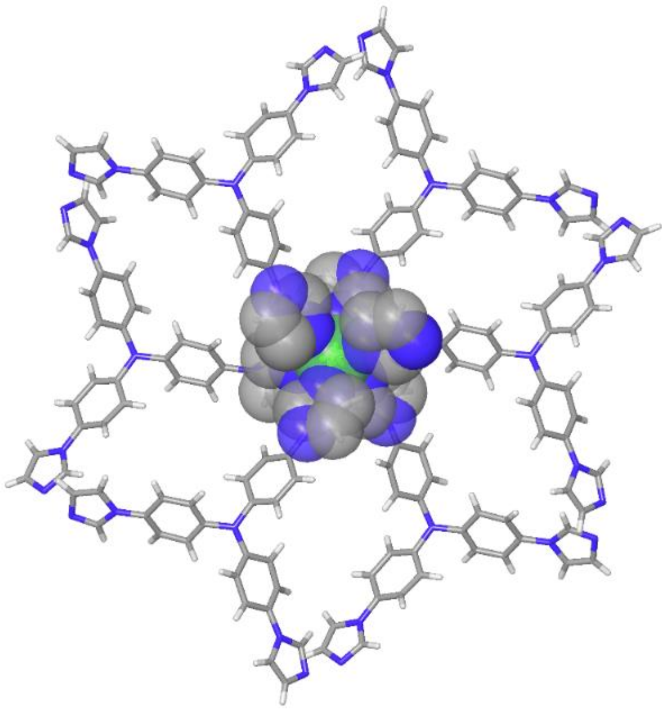

Supplementary Figure 4. Space-filling mode of the coordination environment of a Ni^{2+} ion.

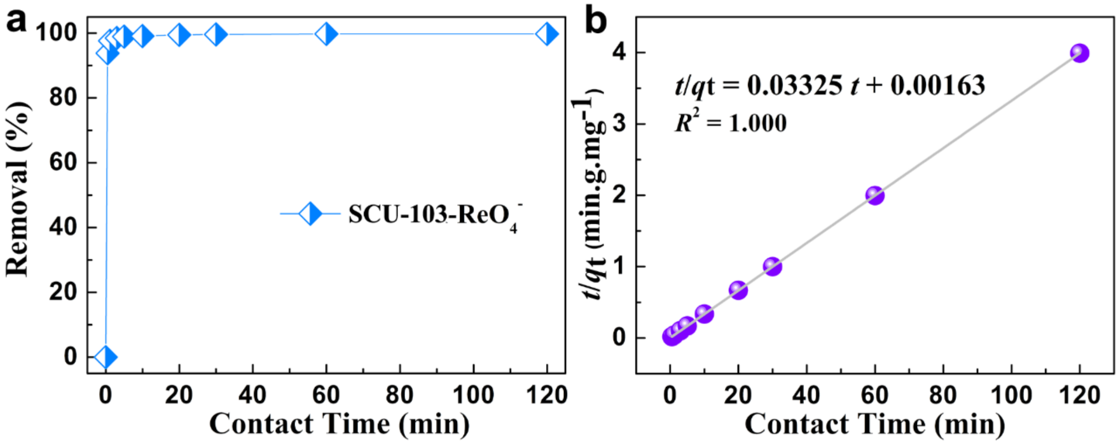

Supplementary Figure 5. (a) Kinetic curve of ReO_4^- ion-exchange using the **SCU-103** plotted as the relative amounts of ReO_4^- removed (%) *vs.* the time *t* (min). (b) The plots of t/q_t *vs.* *t*.

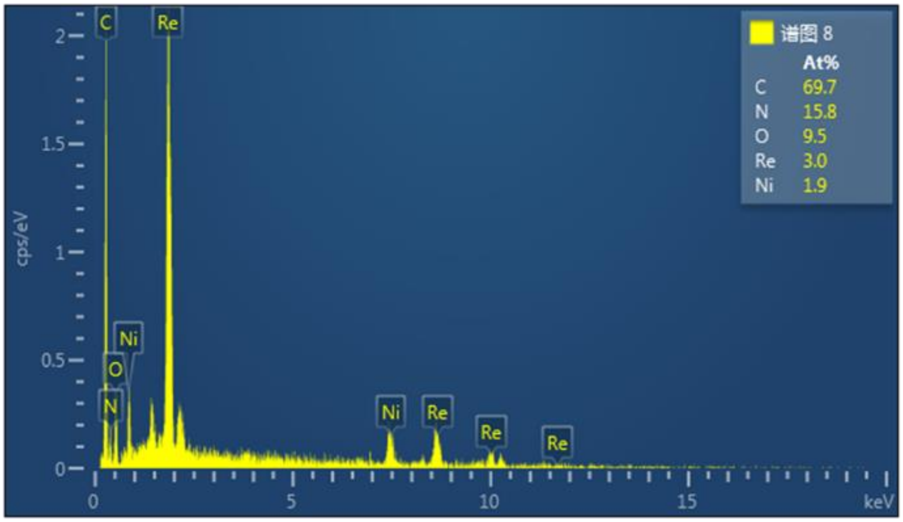

Supplementary Figure 6. EDS diagram of the ReO_4^--exchanged **SCU-103** products.

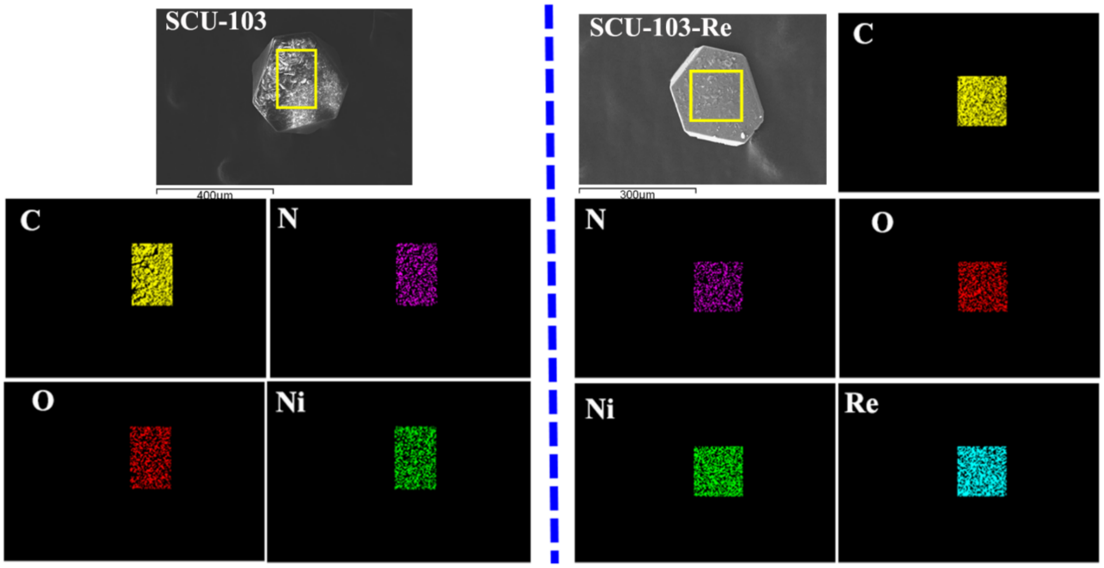

Supplementary Figure 7. EDS mapping of **SCU-103** before and after absorbing ReO_4^-.

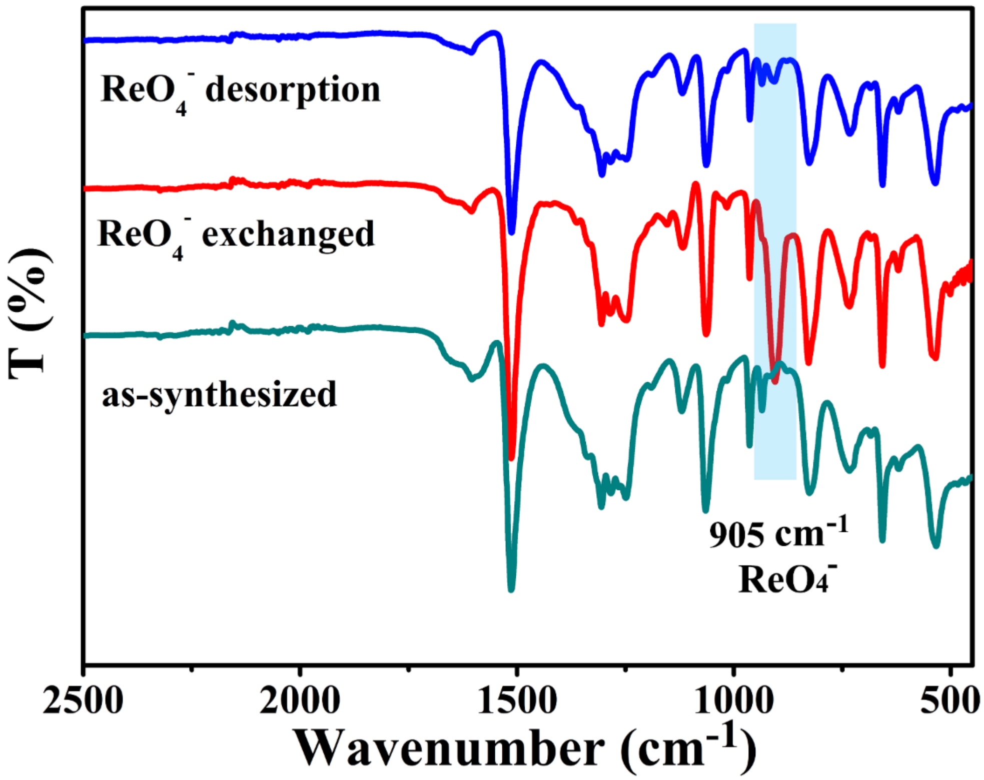

Supplementary Figure 8. FT-IR spectra of the original (green), ReO_4^- exchanged (red) and ReO_4^- desorbed (blue) **SCU-103** products.

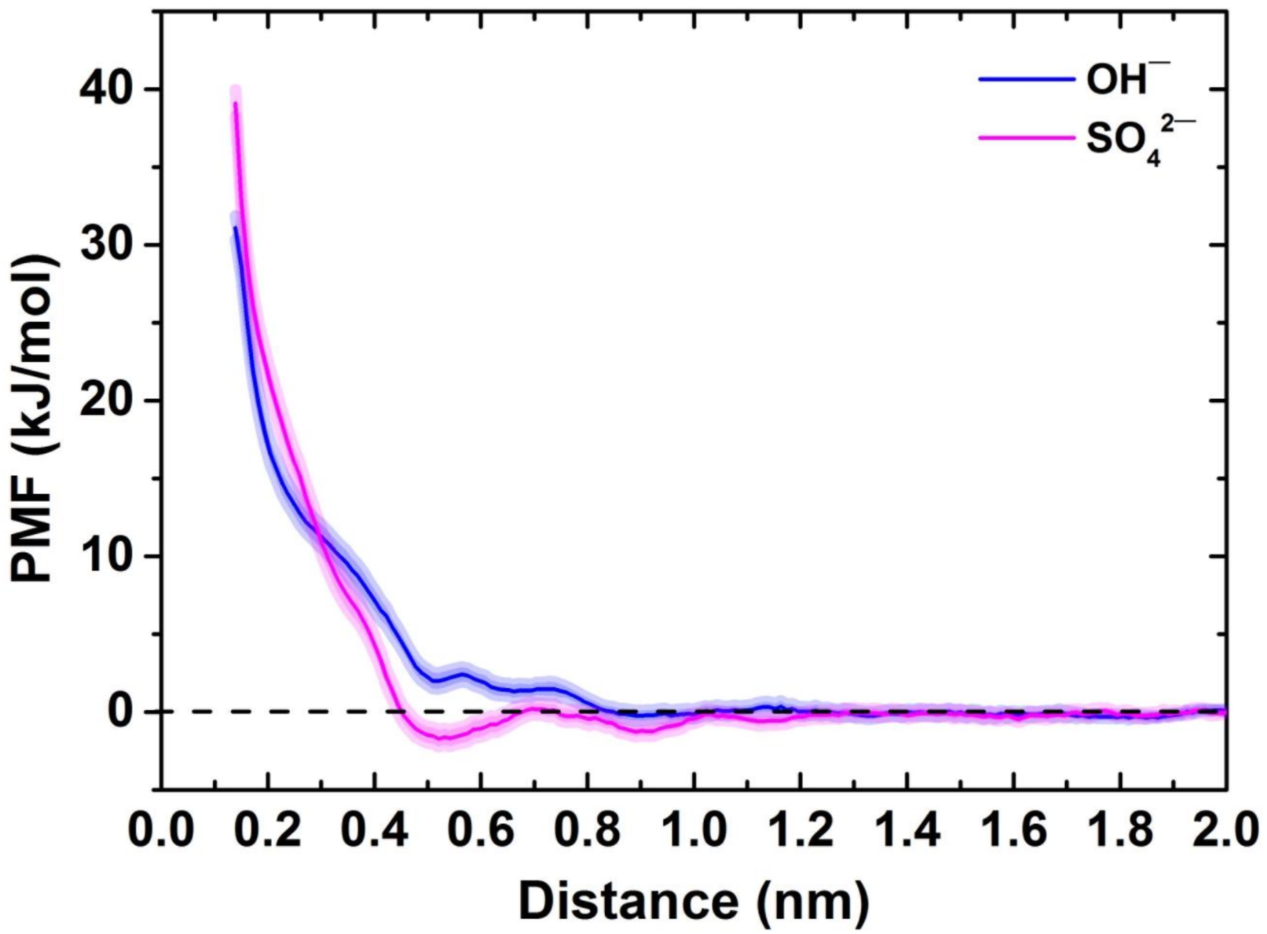

Supplementary Figure 9. Potential of mean forces of single OH^-, and SO_4^{2-} along a reaction coordinate of the center of mass (COM) of the target anion with respected to the COM of **SCU-103**

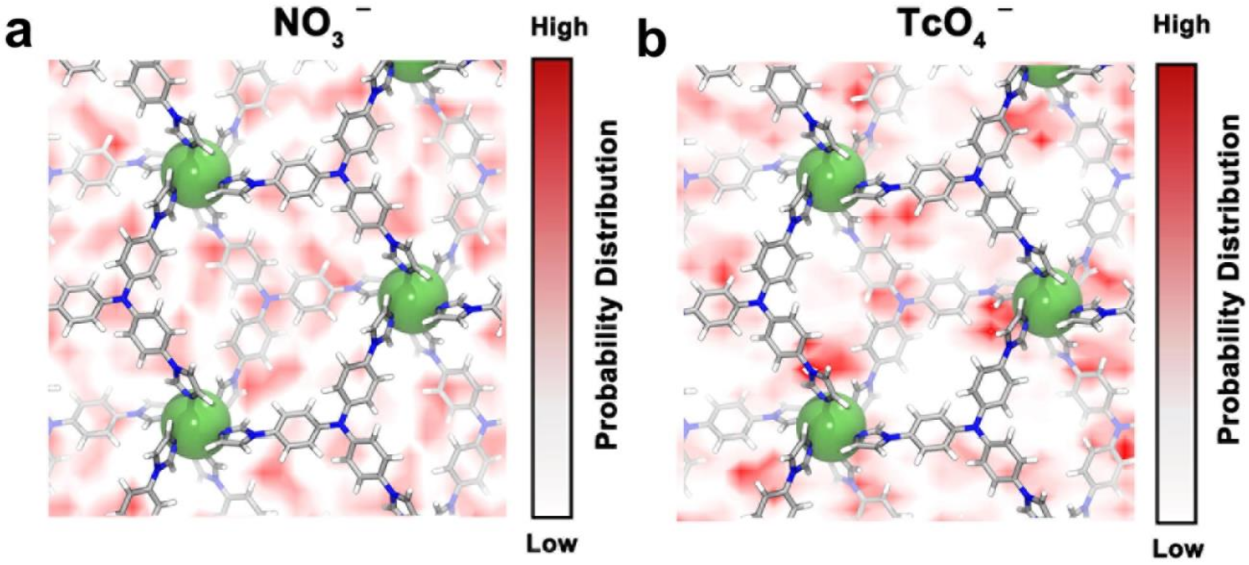

Supplementary Figure 10. The binding probability distributions of (A) NO_3^- and (B) $^{99}TcO_4^-$ to **SCU-103** in the absence of other competing anions. A color scale from lowest probability (0, white) to highest probability (red , 0.110) is used.

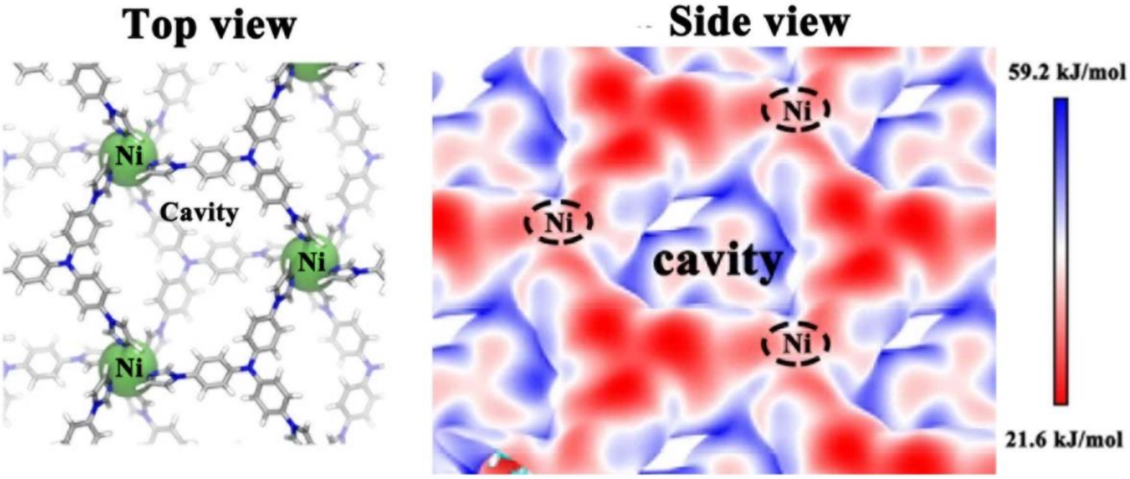

Supplementary Figure 11. The electrostatic potential surface of **SCU-103**, where the blue regions are higher positively charged and red regions are relatively lower positively charged.

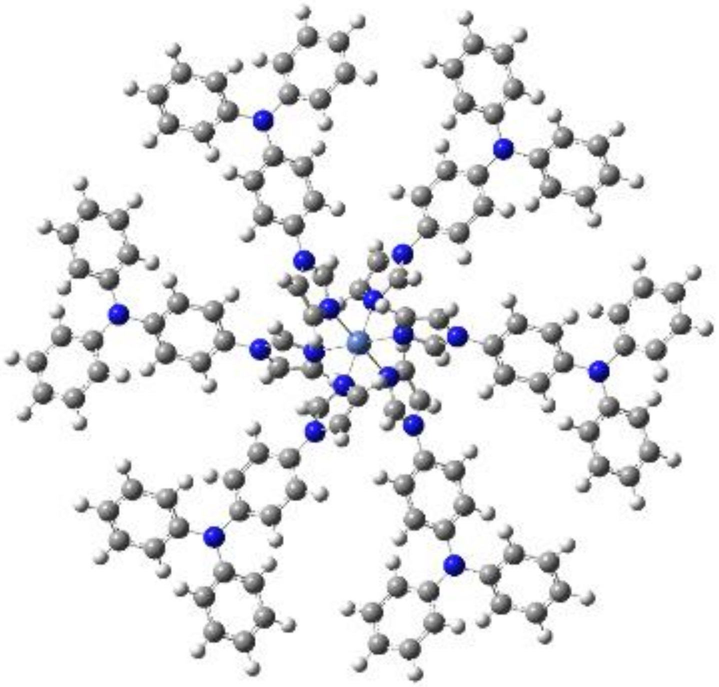

Supplementary Figure 12. The unit cell used in the density functional theory calculations for calculating the atomic charges of the **SCU-103**.

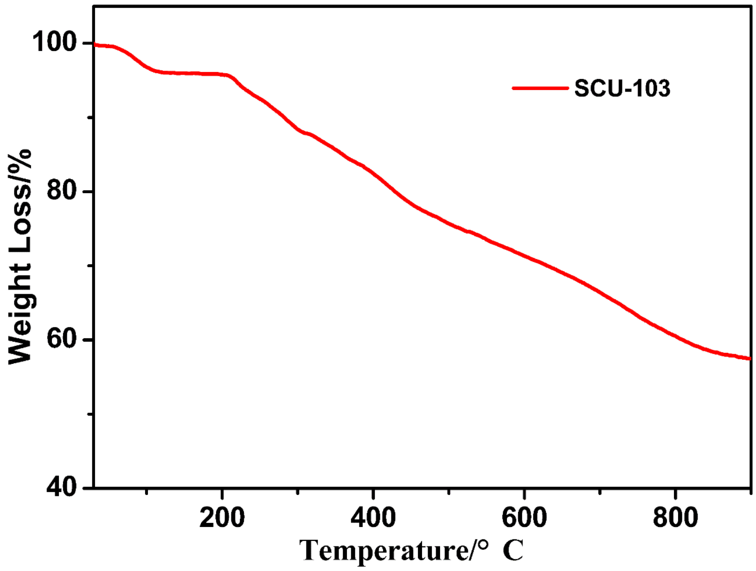

Supplementary Figure 13. The thermogravimetric analysis of SCU-103.

Tables:

Supplementary Table 1. The data for the concentrations of $^{99}TcO_4^-$ at the different times in kinetics experiments.

Time (min)	^{99}Tc activity (CPMA)	Removal (%)
0	95442	0
0.5	7510	92.13
2	2116	97.78
5	1316	98.62
10	1088	98.86

Supplementary Table 2. The data for the concentrations of ReO_4^- (C_t), sorption capacities (q_t) and the relative amounts of ReO_4^- removed (%) at the different times in kinetics experiments.

Time	C_t (ppm)	q_t (mg/g)	Removal (%)
0	40.50	0	0
30 s	2.515	37.98	93.79
70 s	0.9648	39.53	97.61
3 min	0.6087	39.88	98.49
5 min	0.4461	40.05	98.90
10 min	0.3816	40.11	99.06
20 min	0.2150	40.28	99.47
30 min	0.1693	40.32	99.58
60 min	0.1020	40.39	99.74
120 min	0.09446	40.40	99.76

Supplementary Table 3. Exchange capacities for isotherm studies at the different initial ReO_4^- concentrations.

ReO_4^- concentration (ppm)		q_e (mg/g)	K_d (mL/g)
C_0	C_e		
6.799	0.07864	6.720	8.55×10^4
13.45	0.06414	13.39	2.09×10^5
40.49	0.1162	40.38	3.47×10^5
68.43	0.3462	68.08	1.97×10^5
136.4	1.507	134.9	8.95×10^4
205.9	5.066	200.8	3.96×10^4
272.9	11.08	261.8	2.36×10^4
338.3	36.42	301.9	8.29×10^3
419.9	90.19	329.7	3.66×10^3
483.9	168.9	315.0	1.87×10^3
550.2	239.1	311.1	1.30×10^3

Supplementary Table 4. Fitting results from the Langmuir and Freundlich models.

Langmuir			Freundlich		
q_m (mg g^{-1})	K_L (L mg^{-1})	R^2	k_F (L^n/mol^{n-1} g)	n	R^2
318.9	0.461	0.986	117.28	4.799	0.856

Supplementary Table 5. The results for the ReO_4^--exchange of **SCU-103** with individual competitive anions.

**Solution**	**ReO_4^- concentration (ppm)**		**Removal (%)**	**K_d (mL/g)**
	**C_0**	**C_e**		
0.5 mM ReO_4^-	136.4	1.506	98.9	8.95×10^5
0.5 mM ReO_4^-+ 0.5 mM SO_4^{2-}	140.9	2.483	98.2	5.58×10^5
0.5 mM ReO_4^-+ 0.5 mM PO_4^{3-}	155.9	1.698	98.9	9.08×10^5
0.5 mM ReO_4^-+ 0.5 mM NO_3^-	137.2	1.799	98.7	7.52×10^5
0.5 mM ReO_4^-+ 0.5 mM ClO_4^-	177.7	2.016	98.9	8.71×10^5
0.5 mM ReO_4^-+ 0.5 mM CO_3^{2-}	145.1	2.410	98.3	5.92×10^5
0.5 mM ReO_4^-+ 0.5 mM Cl^-	150.8	1.346	99.0	1.02×10^6

Supplementary Table 6. The results for the ReO_4^--exchange of **SCU-103** in the presence of different amounts of NO_3^- ions.

Molar ratio of ReO_4^- and NO_3^-	ReO_4^- (mM)	NO_3^- (mM)	ReO_4^- concentration (ppm)		Removal (%)	K_d (mL/g)
			C_0	C_e		
1:0	0.15	0	40.49	0.1162	99.7	3.47×10^5
1:1	0.15	0.15	40.70	0.1407	99.6	2.88×10^5
1:5	0.15	0.75	35.67	0.2737	99.2	1.29×10^5
1:10	0.15	1.5	35.69	0.4250	98.8	8.30×10^4
1:20	0.15	3	35.47	0.7378	97.9	4.71×10^4
1:100	0.15	15	38.08	4.249	88.8	7.96×10^3

Supplementary Table 7. The results for the ReO_4^--exchange of **SCU-103** in the presence of different amounts of SO_4^{2-} ions.

**Molar ratio of ReO_4^- and SO_4^{2-}**	**ReO_4^- (mM)**	**NO_3^- (mM)**	**ReO_4^- concentration (ppm)**		**Removal (%)**	**K_d (mL/g)**
			**C_0**	**C_e**		
1:0	0.08	0	13.45	0.06414	99.5	2.09×10^5
1:1	0.08	0.08	21.29	0.08668	99.6	2.44×10^5
1:10	0.08	0.8	20.14	0.5831	97.1	3.35×10^4
1:100	0.08	8.0	19.83	0.9051	95.4	2.09×10^4
1:1000	0.08	80.0	18.13	1.966	89.2	8.22×10^3
1:6000	0.08	480.0	17.43	3.081	83.3	4.66×10^3

Supplementary Table 8. ReO_4^- sorption results of **SCU-103** under different pH values.

pH	ReO_4^- concentration (ppm)	q_e (mg/g)	Removal (%)
**Initial**	325.6	**--**	**--**
**3**	29.60	295.9	90.9
**4**	13.29	312.2	95.9
**5**	15.15	310.4	95.3
**6**	15.81	309.7	95.1
**7**	19.03	306.5	94.2
**8**	13.82	311.7	95.8
**9**	13.63	311.9	95.8
**10**	13.56	312.0	95.8
**11**	17.41	308.1	94.7
**12**	34.72	290.8	89.3
**0.1 M NaOH**	126.5	199.1	61.2
**1 M NaOH**	253.1	72.50	22.3

Supplementary Table 9. ReO_4^- sorption results of **SCU-103** in 1M NaOH aqueous solutions at different phase ratios.

**Phase ratio (mg/mL)**	**ReO_4^- concentration (ppm)**	**Removal (%)**
**Blank**	192.7	**--**
**1**	172.5	22.3
**5**	105.3	52.6
**10**	0.8186	99.6
**20**	0.2618	99.9

Supplementary Table 10. Composition of simulated Savannah River Site (SRS) High Level Waste (HLW) Stream.

Anions	Concentration (mol/L)	Anion:TcO_4^- molar ratio
ReO_4^-/TcO_4^-	7.92×10^{-5}	1.0
NO_3^-	2.6	32828
OH^-	1.33	16793
NO_2^-	1.34×10^{-1}	1692
SO_4^{2-}	5.21×10^{-1}	6578
CO_3^{2-}	2.6×10^{-2}	328
$Al(OH)_4^-$	4.29×10^{-1}	5417

Supplementary Table 11. $^{99}TcO_4^-$ sorption results of **SCU-103** by liquid scintillation counting in simulated SRS waste.

Phase ratio (mg/mL)	^{99}Tc activity (CPMA)	Removal (%)
**Blank**	10792	--
**1**	8735	19.0
**5**	5194	51.9
**10**	3787	64.9
**20**	2196	79.7
**40**	1162	89.2

Supplementary Table 12. Chemical composition of the actual SRS tank waste sample used for testing.

Component	Concentration (mol/L)*
Na	5.38
K	7.42×10^{-3}
B	4.15×10^{-3}
Free OH^{-}	1.88
$Al(OH)_4^{-}$	0.177
CO_3^{2-}	0.240
NO_3^{-}	1.82
NO_2^{-}	0.489
SO_4^{2-}	4.00×10^{-2}
Cl^{-}	1.33×10^{-2}
$C_2O_4^{2-}$	6.33×10^{-3}
HCO_2^{-}	4.06×10^{-3}
PO_4^{3-}	6.87×10^{-2}

*Excluded components with concentrations below 0.001 M

Supplementary Table 13. $^{99}TcO_4^-$ sorption results of **SCU-103** by liquid scintillation counting in the actual SRS tank waste.

Phase ratio (mg/mL)	^{99}Tc activity (dpm/mL)	1 sigma unc.	Removal (%)
**Blank**	91700	5.94%	--
**1**	71200	5.78%	22
**5**	43200	5.80%	53
**10**	32500	5.90%	65
**20**	16200	5.76%	82
**40**	9340	5.83%	90

Supplementary Table 14. Results of reusability of **SCU-103** for removing ReO_4^- at pH 7 with a solid-liquid ratio of 1 mg/mL.

		**ReO_4^- concentration (ppm)**	**Removal (%)**	**K_d (mL/g)**
**Cycle numbers**	Initial	36.35		
	1	0.05830	99.8	6.22×10^5
	2	0.5123	98.6	7.00×10^4
	3	0.9298	99.7	3.90×10^5
	4	0.2198	99.4	1.64×10^5

Supplementary Table 15. Results of reusability of **SCU-103** for removing ReO_4^- at pH 14 with a solid-liquid ratio of 10 mg/mL.

		ReO_4^- concentration (ppm)	Removal (%)	K_d (mL/g)
**Cycle numbers**	Initial	247.0		
	1	2.903	98.8	8.41×10^4
	2	18.26	92.6	1.25×10^4
	3	49.05	78.5	3.65×10^4
	4	91.02	60.1	1.51×10^4

Supplementary Table 16. Results of ReO_4^- sorption capacity by **SCU-103** after being irradiated with different doses of γ and β radiations.

		ReO_4^- concentration (ppm)	q_e (mg/g)	Mean (mg/g)	Standard Deviation
	Initial	548.1	--	--	--
**After Sorption**					
γ-ray	100 kGy-dry	204.1	344.0	345.8	6.480
		207.7	340.4		
		195.1	353.0		
	100 kGy-wet	224.5	323.6	322.9	1.335
		226.7	321.4		
		224.4	323.8		
	200 kGy-dry	243.5	304.7	329.8	22.34
		200.6	347.5		
		211.1	337.1		
	200 kGy-wet	222.4	325.8	333.8	7.344
		207.9	340.2		
		212.9	335.3		
	Initial	560.8			
**After Sorption**					
β-ray	100 kGy-dry	239.7	321.1	337.1	13.84
		216.5	344.4		
		215.1	345.8		
	100 kGy-wet	231.3	329.6	324.8	8.433
		245.8	315.1		
		231.1	329.8		
	200 kGy-dry	209.1	351.8	345.8	10.13
		226.8	334.1		
		209.4	351.5		
	200 kGy-wet	253.7	307.1	324.6	15.12
		227.9	333.0		
		227.2	333.7		

Supplementary Table 17. The hydration free energies of various anions obtained by all molecular dynamics simulations here and previous experimental results.

Anions	△G_{sim} (kJ/mol)	Experimental (kJ/mol)[26,27]
OH^{-}	-434.6 ± 0.1	-439
NO_3^{-}	-344.6 ± 0.3	-360
SO_4^{-2}	-1108.4 ± 0.4	-1090
TcO_4^{-}	-263.7 ± 0.2	-251

Movies:

Supplementary Movie S1. Animation of molecular dynamics simulations for the selective sorption of $^{99}TcO_4^-$ into **SCU-103** against various competing anions as depicted in Figure 5A (only $^{99}TcO_4^-$ and NO_3^- anions were shown for clarity).

Supplementary Movie S2. Animation of a representative anion-exchanging process of NO_3^- by $^{99}TcO_4^-$ as depicted in Figure 6A.

References:

1. Jorgensen, W. L. & Tiradorives, J. The opls potential functions for proteins-energy minimizations for crystals of cyclic-peptides and crambin. *J. Am. Chem. Soc.* **110**, 1657-1666 (1988).

2. Williams, C. D. & Carbone, P. A classical force field for tetrahedral oxyanions developed using hydration properties: the examples of pertechnetate (TcO_4^-) and sulfate (SO_4^{2-}). *J. Chem. Phys.* **143**, 174502 (2015).

3. Megyes, T. et al. Solution structure of $NaNO_3$ in water: diffraction and molecular dynamics simulation study. *J. Phys. Chem. B* **113**, 4054-4064 (2009).

4. Bonthuis, D. J., Mamatkulov, S. I. & Netz, R. R. Optimization of classical nonpolarizable force fields for OH^- and H_3O^+. *J. Chem. Phys.* **144**, 104503 (2016).

5. Boudon, S. & Wipff, G. Free-energy calculations involving Nh^{4+} in water. *J.Comput. Chem.* **12**, 42-51 (1991).

6. Abraham, M. J. et al. GROMACS: high performance molecular simulations through multi-level parallelism from laptops to supercomputers. SoftwareX, **1-2**, 19-25 (2015).

7. Humphrey, W., Dalke, A. & Schulten, K. VMD: visual molecular dynamics. *J. Mol. Graphics Modell.* **14**, 33-38 (1996).

8. Berendsen, H. J. C., Grigera, J. R. & Straatsma, T. P. The missing term in effective pair potentials. *J. Phys. Chem.* **91**, 6269-6271 (1987).

9. Bussi, G., Donadio, D. & Parrinello, M. Canonical sampling through velocity rescaling. *J Chem Phys*. **126**, 014101 (2007).

10. Berendsen, H. J. C., Postma, J. P. M., Vangunsteren, W. F., Dinola, A. & Haak, J. R. Molecular-dynamics with coupling to an external bath. *J. Chem. Phys.* **81**, 3684-3690 (1984).

11. Darden, T., York. D. & Pedersen, L. Particle mesh Ewald: an $N{\cdot}\log(N)$ method for Ewald sums in large systems. *J. Chem. Phys.* **98**, 10089-10092 (1993).

12. Hess, B., Bekker, H., Berendsen, H. J. C. & Fraaije, J. G. E. M. LINCS: a linear constraint solver for molecular simulations. *J. Comput. Chem.* 18, 1463-1472(1997).

13. Zacharias, M., Straatsma, T. P. & McCammon, J. A. Separation-shifted scaling, a new scaling method for Lennard-Jones interactions in thermodynamic integration. *J. Chem. Phys.* **100**, 9025-9031 (1994).

14. Kumar, S., Bouzida, D., Swendsen, R. H., Kollman, P. A. & Rosenberg, J. M. The weighted histogram analysis method for free-energy calculations on biomolecules. 1. The method. *J. Comput. Chem.* **13**, 1011-1021 (1992).

15. Hub, J. S., de Groot, B. L. & van der Spoel, D. (2010) g_wham-A free weighted histogram analysis implementation including robust error and autocorrelation estimates. *J. Chem. Theory Comput.* **6**, 3713-3720 (2010).

16. Efron, B. Bootstrap methods: another look at the jackknife. Ann. Statist. **7**, 1-26 (1979).

17. Delley, B. An all-electron numerical-method for solving the local density functional for polyatomic-molecules. *J. Chem. Phys.* **92**, 508-517 (1990).

18. Delley, B. From molecules to solids with the DMol(3) approach. *J. Chem. Phys.* **113**, 7756-7764 (2000).

19. Perdew, J. P. Burke, K. & Ernzerhof, M. Generalized gradient approximation made simple. *Phys. Rev. Lett.* **77**, 3865-3868 (1996).

20. Frisch, M. J. et al. Gaussian 09, Revision E.01) (2009).

21. Becke, A. D. Density-functional thermochemistry. 3. The role of exact exchange. *J. Chem. Phys.* **98**, 5648-5652 (1993).

22. Lee, C. T., Yang, W. T. & Parr, R. G. Development of the colle-salvetti correlation-energy formula into a functional of the electron-density. *Phys. Rev. B* **37**, 785-789 (1988).

23. Hehre, W. J., Ditchfie, R. & Pople, J. A. Self-consistent molecular-orbital methods. 12. Further extensions of gaussian-type basis sets for use in molecular-orbital studies of organic-molecules. *J. Chem. Phys.* **56**, 2257-2261 (1972).

24. Scalmani, G. & Frisch, M. J. Continuous surface charge polarizable continuum models of solvation. I. General formalism. *J. Chem. Phys.* **132**, 114110 (2010).

25. Breneman, C. M. & Wiberg, K. B. Determining atom-centered monopoles from molecular electrostatic potentials. The need for high sampling density in formamide conformational analysis. *J. Comput. Chem.* **11**, 361-373 (1990).

26. Custelcean, R. & Moyer, B. Anion separation with metal organic frameworks. *Eur. J. Inorg. Chem.* **2007**, 1321-1340 (2007).

27. Marcus, Y. Thermodynamics of solvation of ions. Part 5.-Gibbs free energy of hydration at 298.15 K. *J. Chem. Soc., Faraday Trans.* **87**, 2995-2999 (1991).
